# Dual‐Strategy Design of Heterojunction‐Enhanced Piezoelectric Hydrogels for Periodontitis Treatment

**DOI:** 10.1002/advs.202600017

**Published:** 2026-02-13

**Authors:** Qiangqiang Zhou, Shaojun Fang, Changyi Li, Mengqi Zhou, Xin Sui, Chen Hu, Huaxing Xu, Shiyi Yang, Bing‐Qiang Lu, Rongjun Zhang, Xiaoling Wei

**Affiliations:** ^1^ Department of Endodontics, Shanghai Stomatological Hospital and School of Stomatology Fudan University Shanghai China; ^2^ Shanghai Key Laboratory of Craniomaxillofacial Development and Diseases Fudan University Shanghai China; ^3^ Key Laboratory of Micro and Nano Photonic Structures (MOE), Shanghai Engineering Research Center of Ultra‐Precision Optical Manufacturing, College of Future Information Technology Fudan University Shanghai China

**Keywords:** heterojunction, piezoelectric hydrogel, periodontal regeneration, osteoimmune modulation, ultrasound stimulation

## Abstract

Periodontitis is a widespread chronic inflammatory disease that threatens oral and systemic health by sustaining inflammation and accelerating alveolar bone loss. Although bioelectrical modulation can promote bone repair under inflammatory conditions, the coordinated regulation of electrical signaling, immunoregulation, and osteogenesis within an inflamed microenvironment remains a central challenge. Here, we report a multifunctional piezoelectric hydrogel system that combines heterojunction enhancement with dual‐salt synergy. In situ construction of ZnO/ZnS heterojunctions with oxygen vacancies amplifies the piezoelectric output of ZnO, while a polyvinyl alcohol (PVA) multinetwork integrated with magnesium chloride (MgCl_2_) and trisodium citrate (Na_3_Ct) provides high ionic conductivity, mechanical robustness, and bioactivity, conferring excellent injectability and environmental adaptability. The composite hydrogel exhibited low impedance and high ionic conductivity (≈3.82 mS·cm^−^^1^), generated a stable voltage of ≈150 mV under ultrasound activation, and maintained sensitive strain response under moist conditions. In vitro, the hydrogel markedly enhanced osteogenic differentiation of human periodontal ligament stem cells (hPDLSCs), with upregulation of Runx2, Col‐1, OPN, and OCN, and simultaneously drove macrophage polarization toward the M2 phenotype. A conditioned medium model further confirmed immune remodeling that alleviated the inflammatory burden of hPDLSCs. In a rat periodontitis model, the hydrogel restored alveolar bone architecture, reduced osteoclast activity, and rebalanced the M1/M2 ratio. Collectively, this piezoelectric hydrogel enhanced by heterojunctions provides a versatile platform for immunologically instructive regeneration of inflamed bone defects.

## Graphical abstract

This study developed a piezoelectric hydrogel integrating heterojunction enhancement and dual‐salt synergy, combining robust electrical performance with mechanical stability and bioactivity to better match the periodontal microenvironment. Under ultrasound activation, the hydrogel markedly promotes macrophage M2 polarization, thereby reducing the inflammatory burden of hPDLSCs and accelerating alveolar bone regeneration. This work offers a translationally promising bioelectric platform for periodontitis therapy.

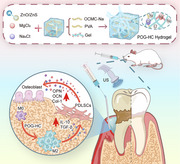

## 1 Introduction

Periodontitis is a common oral disease characterized by chronic inflammation and progressive tissue destruction, affecting over 60% of the global population and resulting in more than one billion severe cases [1, 2]. Its persistent inflammatory state is closely associated with systemic comorbidities such as diabetes and cardiovascular diseases, posing substantial risks to both oral and general health [3]. Yet current clinical and laboratory interventions rarely achieve concurrent inflammation control and regeneration, leading to high recurrence rates [4]. Therefore, therapeutic strategies that directly address the osteoimmune imbalance in periodontitis are urgently needed to shift the paradigm from mere lesion control to regenerative repair. Given that periodontitis is frequently accompanied by marked alveolar bone resorption (an inflammatory bone defect), restoration of bone tissue becomes a central goal.

Bioelectric modulation has emerged as a front‐line approach that mimics endogenous cues to regulate stem cell fate, tissue reconstruction, and immune homeostasis [5, 6, 7]. In inflammatory bone defects, exogenous electrical stimulation can break the inflammation‐induced osteoinhibitory feedback loop and reprogram the regenerative microenvironment [8, 9]. Importantly, bone is intrinsically piezoelectric [10], endowing the alveolar bone with unique mechano‐electrophysiological coupling [11, 12] and thereby offering natural compatibility for electrical interventions. Recent wireless and non‐invasive piezoelectric materials, including BaTiO_3_, ZnO, and PVDF, have shown promise for bone regeneration and inflammation control [13, 14, 15]. However, an ideal periodontal piezoelectric material system must meet four criteria simultaneously: (i) robust piezoelectric response; (ii) efficient electric‐field conduction; (iii) strong adaptability to the complex lesion niche; and (iv) coordinated immuno‐osteogenic effects. Current materials still face application‐specific limitations in the oral cavity—e.g., PVDF offers flexibility but lacks osteoconductivity and bioactivity; BaTiO_3_ exhibits a high piezoelectric constant but is excessively stiff for soft tissue interfaces, while improvements along a single dimension often fail to address clinical complexity [16]. Thus, synergistic material designs are essential for clinical transition of bioelectric strategies [17].

Among piezoelectric candidates, ZnO stands out for its biocompatibility and synthetic controllability [18]. Nevertheless, conventional ZnO nanomaterials exhibit limited piezoelectric performance; bulk free carriers can migrate under the piezoelectric field and form a screening layer that neutralizes polarized charges, yielding weak and unstable outputs [19]. Heterojunction engineering offers an effective solution: the built‐in electric field can cooperate with the piezoelectric field to enhance charge separation and directed transport while suppressing interfacial carrier accumulation, thereby amplifying the output [20, 21]. In parallel, interfacial defect engineering introduces electron traps that capture free carriers and facilitate ordered migration, further boosting performance [22, 23]. However, ex situ synthesis strategies and structural differences often lead to lattice mismatch, increase interfacial impedance, and result in performance degradation. In this study, an in situ construction strategy was employed to prepare ZnO/ZnS heterojunction materials with excellent lattice matching. The heterointerface could facilitate efficient electron transport and mitigate the shielding effect. Meanwhile, the substitution of sulfur atoms for oxygen atoms and the annealing process would introduce interfacial oxygen vacancies (Ov), which synergize with the piezoelectric effect of ZnS to further enhance carrier migration, thus achieving a *two‐pronged* enhancement via heterojunction plus defect engineering.

However, advancing piezoelectric materials alone is insufficient. A parallel, equally crucial challenge lies in the stable and efficient delivery of the generated electrical cues to the confined periodontal lesion [24]. This necessitates a delivery vehicle that must not only ensure robust electrical conduction but also physically adapt to the complex, inflamed microenvironment. PVA‐based hydrogels, with their 3D networks, biocompatibility, degradability, and tissue adhesion, are widely used for local delivery and tissue engineering [25]. Although PVA is attractive as a base matrix, its intrinsic low conductivity limits electrical field transfer, and its injectability and crosslink tunability in narrow, irregular pockets still require improvement. Recent evidence indicates that specific Hofmeister‐series ions can enhance ionic conductivity and modulate PVA interchain hydrogen bonding, synergistically improving mechanics and charge transport. Accordingly, we proposed a *one‐stone‐multi‐birds* dual‐salt system in a PVA matrix using MgCl_2_ and Na_3_Ct to increase ionic conductivity and refine the network via multivalent coordination and ionic regulation, thereby achieving injectability, periodontal adaptability, and efficient electrical conduction [26]. Notably, Mg^2^^+^ can further steer macrophage polarization and activate osteogenic signaling, conferring dual functions in immunomodulation and bone regeneration [27, 28, 29].

**SCHEME 1:**
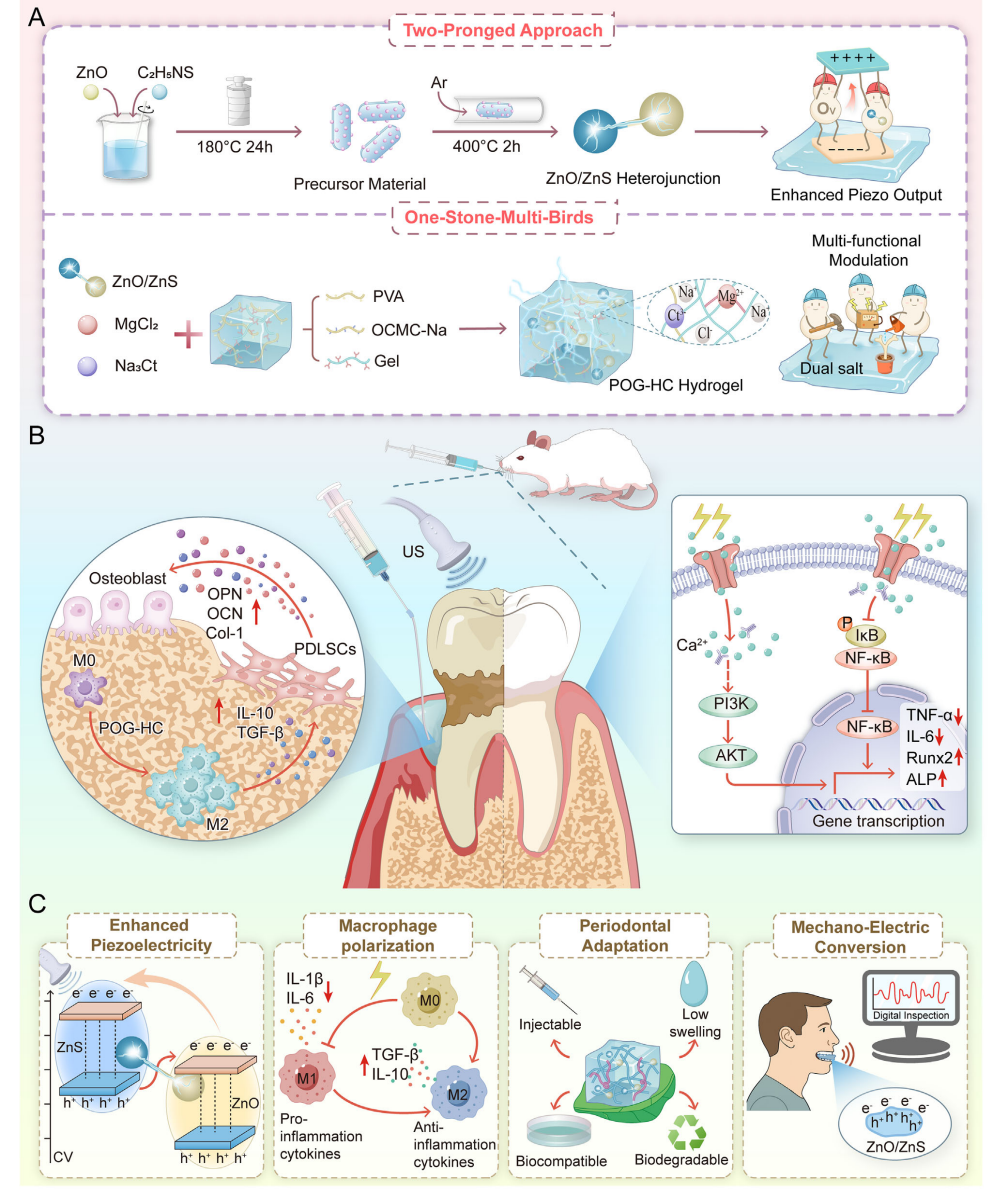
Piezoelectric hydrogel with integrated dual‐strategy design for periodontal therapy. (A) *Two‐Pronged Approach*: ZnO piezoelectricity is enhanced by combining heterojunction formation and oxygen vacancy engineering. *One‐Stone‐Multi‐Birds Strategy*: Dual salts (MgCl_2_/Na_3_Ct) reinforce the hydrogel by improving mechanical strength, ionic conductivity, and osteoinductive delivery. (B,C) The hydrogel facilitates osteoimmune regeneration while offering improved piezoelectric output, immune modulation, and tissue adaptability.

In summary, we synergistically integrate Ov‐rich ZnO/ZnS piezoelectric heterojunction particles into a dual‐salt modulated conductive hydrogel matrix (Scheme 1). This multinetwork composite hydrogel simultaneously enhances piezoelectric output and optimizes mechanics, conductivity, and injectability, enabling coordinated regulation of mechanics‐electricity‐immunity within the periodontitis niche. We thereby target concurrent osteogenesis promotion and immune remodeling at the lesion site, offering an engineered and bioadaptive pathway for regenerative therapy of periodontitis.

## 2 Results and Discussion

### 2.1 Construction of ZnO/ZnS Heterojunctions

**FIGURE 1:**
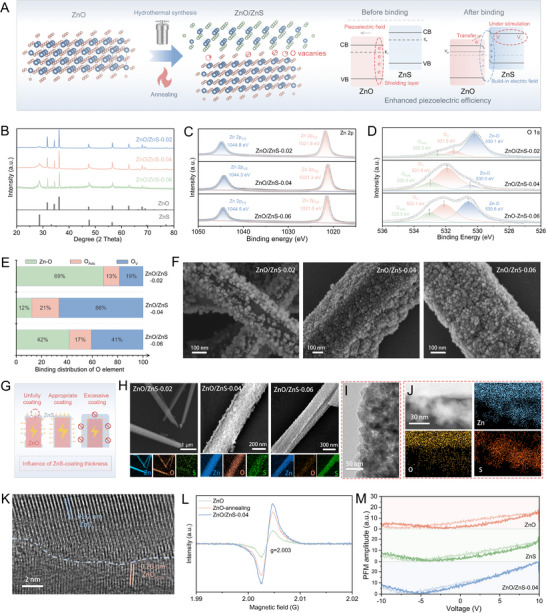
Construction of ZnO/ZnS heterojunction and characterization of their structure and piezoelectric performance. (A) Schematic illustration of the ZnO/ZnS synthesis and the piezoelectric enhancement mechanism. (B) XRD patterns of ZnO/ZnS synthesized with varying sulfur contents. (C,D) XPS spectra (Zn 2p and O 1s) of ZnO/ZnS with different sulfur contents. (E) Oxygen coordination ratios derived from O 1s spectra at different sulfur levels. (F) SEM images of ZnO/ZnS nanorods with different sulfur contents (scale bar: 100 nm). (G) Schematic showing the influence of ZnS shell thickness on piezoelectric performance. (H) Elemental mapping images of ZnO/ZnS with varying sulfur contents. (I) TEM image of ZnO/ZnS‐0.04. (J) Elemental mapping of ZnO/ZnS‐0.04. (K) HRTEM image of ZnO/ZnS‐0.04 showing lattice fringes of ZnO and ZnS (scale bar: 2 nm). (L) EPR spectra of ZnO/ZnS‐0.04, pure ZnO, and annealed ZnO. (M) PFM curves comparing the piezoelectric response of ZnO/ZnS‐0.04, pure ZnO, and pure ZnS.

A *two‐pronged strategy* was employed to modify ZnO and synergistically enhance its piezoelectric performance (Figure 1A). In pure ZnO, the excited electrons tend to migrate under the internal piezoelectric field and accumulate at the positive end, forming a screening layer that weakens the net piezoelectric output of the material [30]. In contrast, the ZnO/ZnS heterojunction forms a type‐II band alignment at the interface due to the energy level difference between ZnO and ZnS, generating a built‐in electric field that directs electrons from ZnO toward ZnS. This alleviates interfacial charge accumulation and mitigates the screening effect [31]. Moreover, sulfur substitution of oxygen and the annealing process would induce oxygen vacancies at the interface [32], which act as electron traps and facilitate charge transport [33]. Furthermore, ZnS itself also exhibits intrinsic piezoelectricity, contributing to improved charge separation [34]. Together, these mechanisms offer a multidimensional enhancement of ZnO's piezoelectric performance.

To optimize the component ratio, a series of ZnO/ZnS‐x samples was synthesized with varying sulfur precursor contents (x = 0.02, 0.04, 0.06, 0.08). X‐ray diffraction (XRD) patterns (Figure 1B) revealed coexisting diffraction peaks of ZnO and ZnS in ZnO/ZnS‐0.02 to ZnO/ZnS‐0.06, with increasing ZnS intensity as sulfur content rose. However, ZnO/ZnS‐0.08 exhibited near complete transformation to ZnS, suggesting that the heterojunction was successfully fabricated mainly in the ZnO/ZnS‐x (x = 0.02, 0.04, 0.06) samples (Figure S1). X‐ray photoelectron spectroscopy (XPS) spectra further showed that the Zn 2p binding energy in ZnO/ZnS‐0.04 shifted to lower values compared to low sulfur samples, suggesting increased interfacial oxygen vacancies and electron enrichment around Zn atoms. With excessive sulfur, the peak shifted back to higher binding energies, indicating that excessive sulfur fills oxygen vacancies and withdraws electrons (Figure 1C; Figure S2). The O 1s spectrum confirmed that ZnO/ZnS‐0.04 had the highest oxygen vacancy ratio (Figure 1D,E), supporting the above analysis. Moreover, scanning electron microscopy (SEM) images of different ZnO/ZnS samples (Figure 1F) show that all retained the nanorod morphology of ZnO with a surface ZnS shell. ZnO/ZnS‐0.02 displayed incomplete coverage, while ZnO/ZnS‐0.04 and 0.06 exhibited more uniform shells, with 0.06 being thicker. As illustrated in Figure 1G, an appropriate ZnS content is critical for optimal piezoelectric enhancement: insufficient ZnS fails to form a functional heterojunction, while excessive ZnS may eliminate oxygen vacancies and block ZnO's internal electric field [35]. Elemental mapping (Figure 1H) further confirmed that ZnO/ZnS‐0.04 exhibited the most uniform O and S distributions, forming an ideal heterojunction structure. Based on the combined structural and defect analyses, ZnO/ZnS‐0.04 was selected for subsequent studies.

To further characterize the interface structure, transmission electron microscopy (TEM) and elemental mapping of ZnO/ZnS‐0.04 (Figure 1I,J) were tested and the results revealed a distinct ZnS coating at ZnO surface. Zn was uniformly distributed throughout both core and shell regions, O was mainly localized within the core, and S was concentrated on the outer shell, further confirming successful heterojunction formation. High‐resolution transmission electron microscopy (HRTEM) images (Figure 1K) showed well‐defined lattice fringes of both ZnO and ZnS, indicating a typical heterostructure interface. Electron paramagnetic resonance (EPR) results demonstrated the strongest oxygen vacancy signal in ZnO/ZnS‐0.04, demonstrating that the regulation of oxygen vacancies is a result of the synergistic effect between annealing and the in situ sulfidation process, wherein the S substitution further promotes the generation of oxygen vacancies (Figure 1L). Meanwhile, piezoresponse force microscopy (PFM) measurements also revealed the highest piezoelectric response in ZnO/ZnS‐0.04, surpassing that of both pure ZnO and ZnS (Figure 1M; Figure S4). Collectively, these results demonstrate that the in situ constructed ZnO/ZnS‐0.04 heterojunction achieves significant improvement in piezoelectric performance via synergistic interfacial coupling and oxygen vacancy engineering. This provides a solid foundation for its application in periodontal therapy by enabling efficient bioelectrical signal output.

### 2.2 Design and Functional Characterization of Injectable POG‐HC Hydrogel

**FIGURE 2:**
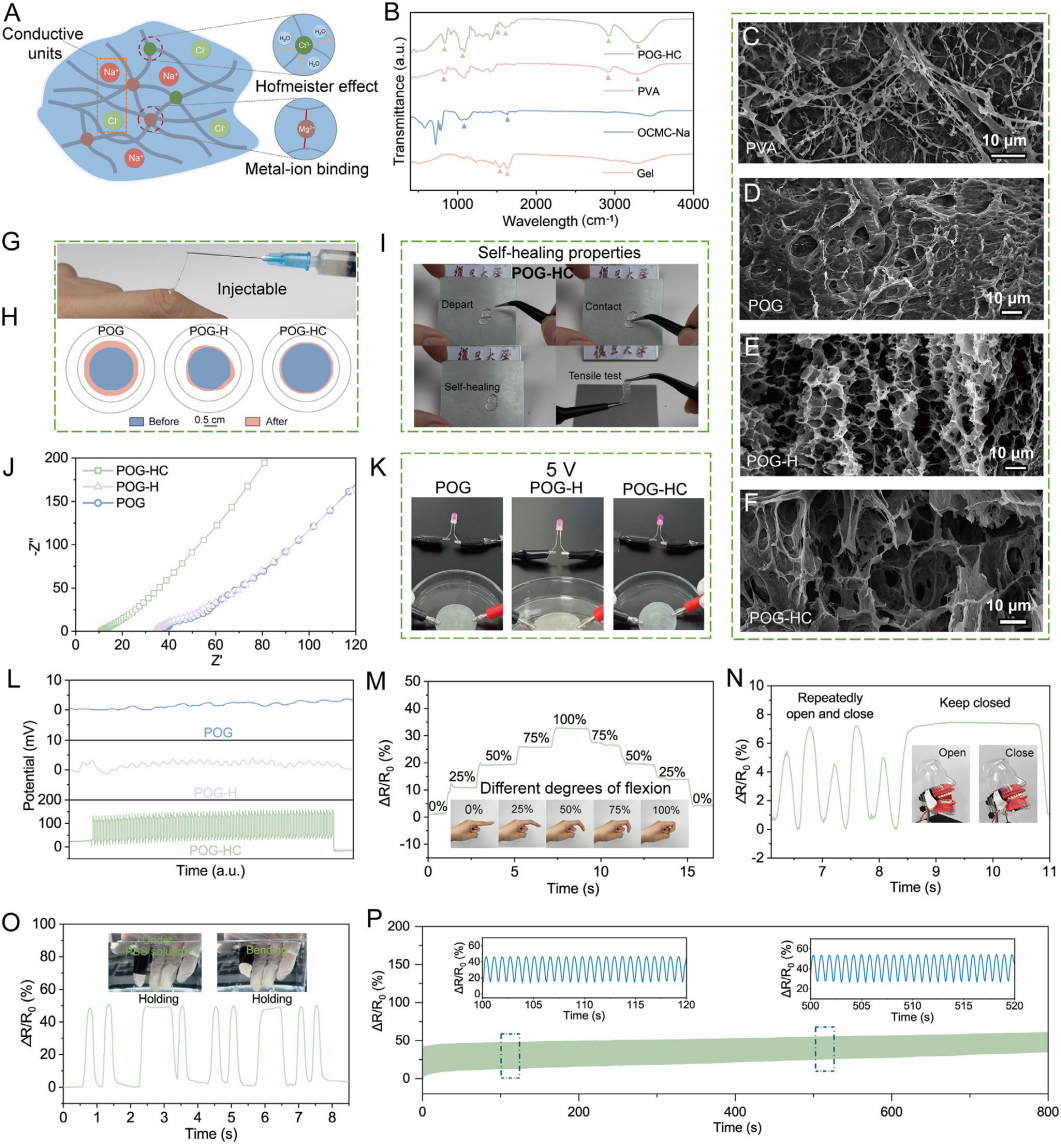
Fabrication and properties of the piezoelectric hydrogel. (A) Schematic illustration of the dual‐salt modulated strategy in the POG‐HC hydrogel. (B) FTIR spectra of POG‐HC and its main components. (C–F) SEM images of PVA, POG, POG‐H, and POG‐HC hydrogels (scale bar: 10 µm). (G) Digital photograph demonstrating the injectability of POG‐HC. (H) Comparison of swelling between POG‐HC, POG‐H, and POG. (I) Self‐healing performance of POG‐HC. (J) EIS curves of POG‐HC, POG‐H, and POG. (K) Conductivity comparison between POG‐HC, POG‐H, and POG. (L) Voltage‐time (V‐t) curves under US stimulation for POG‐HC, POG‐H, and POG. (M) Sensing performance of POG‐HC during finger bending. (N) Sensing performance of POG‐HC under a dental occlusion model. (O) Sensing performance of POG‐HC in PBS solution. (P) Long‐term signal stability of POG‐HC over 1000 cycles. ΔR/R_0_ (%) denotes the normalized resistance change, where ΔR = R − R_0_, R is the instantaneous resistance under stimulation, and R_0_ is the initial resistance in the unstimulated state.

In this study, the *one‐stone‐multi‐birds* strategy was employed by co‐regulating the PVA‐based hydrogel using a dual‐salt system consisting of MgCl_2_ and Na_3_Ct (Figure 2A). The dissociation of salts significantly enhanced the ionic conductivity of the system, which benefits piezoelectric signal transmission [36]. Meanwhile, a synergistic regulation between the Ct^3−^ “salting‐out” and Cl^−^ “salting‐in” effects was implemented, thereby endowing the material with an appropriate injection strength [37]. Additionally, Mg^2^^+^ coordinated with the carboxyl groups of Oxidized Carboxymethylcellulose Sodium (OCMC‐Na) and enabled sustained local release, acting as a bioactive ion to promote periodontal regeneration [38]. This dual‐salt design synergistically improves the hydrogel's conductivity, mechanical stability, and biological activity.

Fourier‐transform infrared spectroscopy (FTIR) spectra (Figure 2B; Figure S3) confirmed the formation of a PVA/Gel/OCMC‐Na multinetwork, in which OCMC‐Na, containing both aldehyde and carboxyl groups, can form Schiff base bonds with gelatin and coordinate with Mg^2+^, enhancing the stability of the hydrogel network. SEM images (Figure 2C–F) showed that POG hydrogel exhibited a dense cross‐linked morphology, while the introduction of the ZnO/ZnS heterojunction (named as POG‐H) resulted in enlarged pores. The pore size was further amplified in the POG‐HC hydrogel (with further addition of dual salts) with the addition of the dual‐ion salt, promoting injectability, ion transport, and cell infiltration. Owing to this rational design, POG‐HC demonstrated excellent injectability and optical transparency (Figure 2G; Figure S5), allowing minimally invasive application in periodontal defects. The hydrogel exhibited a low swelling ratio (∼12%) over 48 h (Figure 2H), which ensures dimensional stability under moist oral conditions. Furthermore, reversible Schiff base bonding endowed POG‐HC with self‐healing properties (Figure 2I), enhancing its environmental adaptability and long‐term functional stability.

Efficient electrical conduction is critical for piezoelectric therapy. Electrochemical impedance spectroscopy (EIS) analysis indicated that POG‐HC exhibited an ionic conductivity of 3.82 mS·cm^−^^1^, significantly higher than that of the other two groups (Figure 2J). Consistently, in a practical circuit test, POG‐HC lit the bulb more brightly under a 5 V supply (Figure 2K), indicative of reduced electrical resistance, and corresponds well with the EIS results. Under ultrasound (US) stimulation, piezoelectric output measurements (Figure 2L) showed that POG‐HC generated a voltage fluctuation of ≈150 mV, markedly higher than POG‐H and POG, confirming the enhanced signal transmission capability conferred by the dual‐salt system.

Owing to its low impedance and high conductivity, the POG‐HC hydrogel exhibits sensitive resistance changes in response to strain stimuli, enabling it to monitor various motions. During finger‐bending tests, it could distinguish subtle changes in bending angle (Figure 2M); in simulated biting motion experiments, the hydrogel produced clear signal differentiation between open and closed jaw states (Figure 2N). Notably, even in PBS‐simulated wet oral conditions, POG‐HC maintained stable signal detection during repeated bending (Figure 2O). Furthermore, the electric signal remained consistent after >1000 cycles of tensile deformation, demonstrating its excellent long‐term durability (Figure 2P). In summary, the multinetwork hydrogel architecture integrated with a dual‐salt system imparts POG‐HC with high conductivity, mechanical resilience, and sensitive piezoelectric responsiveness. These properties enable effective delivery of electric fields to the periodontal defect region, aligning with the therapeutic demands of periodontitis.

### 2.3 Biosafety Evaluation of the Piezoelectric Hydrogel Both In Vitro and In Vivo

**FIGURE 3:**
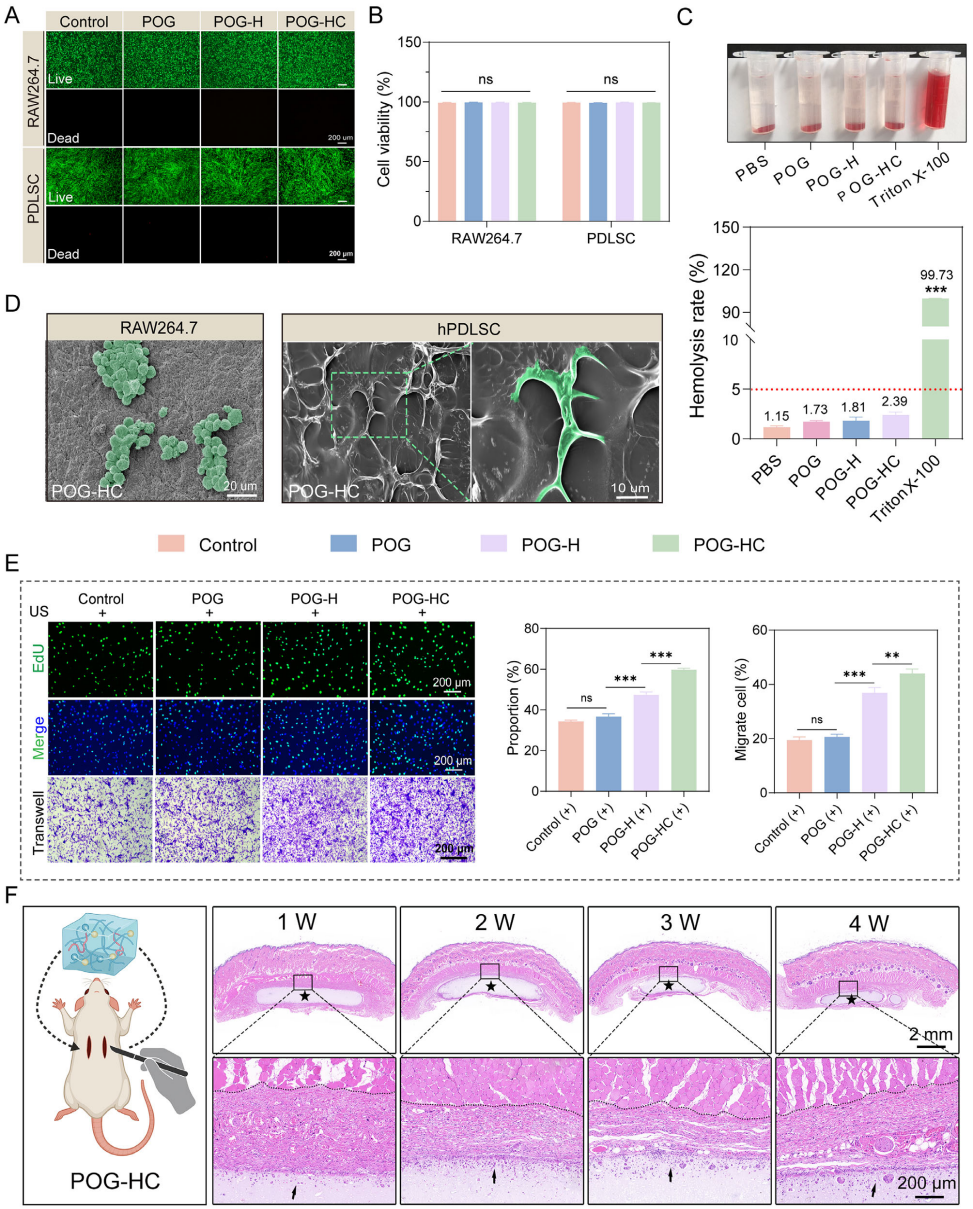
In vitro and in vivo biosafety evaluation of the piezoelectric hydrogel system. (A,B) Live/Dead staining of RAW264.7 macrophages and hPDLSCs treated with various hydrogels to assess cytotoxicity (n=4; scale bar: 200 µm). (C) Hemolysis ratio of each hydrogel group (n = 4). (D) SEM images showing the adhesion morphology of hPDLSCs and RAW264.7 cells on the surface of the POG‐HC hydrogel. (E) EdU and Transwell assays evaluating hPDLSC proliferation and migration under US stimulation across different material groups (n = 4; scale bar: 200 µm). (F) In vivo subcutaneous implantation of the POG‐HC hydrogel, followed by histological analysis to assess material degradation and local inflammatory responses. Data are presented as mean ± SD; ^*^ *p* < 0.05, ^**^ *p* < 0.01, ^***^ *p* < 0.001, ns: not significant.

To assess the biosafety of the piezoelectric hydrogel system, we systematically evaluated its effects on cellular viability, hemocompatibility, and in vivo tissue response. As shown in Figure 3A,B, no evident cytotoxicity was observed in either RAW264.7 macrophages or hPDLSCs upon exposure to POG, POG‐H, or POG‐HC hydrogels. Live/Dead staining confirmed robust cell viability across all groups, and quantitative analyses revealed no significant differences in cell survival (*p* > 0.05), indicating excellent in vitro cytocompatibility. Hemocompatibility was further assessed via hemolysis assays (Figure 3C), where the POG‐HC group exhibited a hemolysis rate of only 2.39%, well below the internationally accepted safety threshold of 5% [ASTM: F756‐17], underscoring its blood compatibility. SEM revealed distinct adhesion behaviors on the POG‐HC hydrogel surfaces (Figure 3D): RAW264.7 cells displayed clustered adhesion, while hPDLSCs exhibited well‐spread morphologies with numerous filopodia tightly anchored to the matrix, suggesting that the POG‐HC hydrogel provides a favorable microenvironment for cell attachment. To probe functional cellular responses, we employed EdU proliferation and Transwell migration assays under US stimulation (Figure 3E). Compared to POG and blank control groups, both POG‐H and POG‐HC significantly enhanced hPDLSC proliferation and migration, with POG‐HC achieving the most pronounced effects (*p* < 0.001).

Considering that the released inorganic ions from the MgCl_2_/Na_3_Ct network may independently influence cell behaviors, we quantified Mg^2+^ and Na^+^ release from the hydrogels in PBS. Both the POG‐HC and POG‐C hydrogels (dual‐salt network without the ZnO/ZnS heterojunction) exhibited comparable release kinetics, indicating that incorporation of the heterojunction did not substantially alter ion release. Based on the release profiles and the extraction conditions used in this study, the terminal Mg^2+^ concentration in the medium was estimated to be 2.1 mM, which falls within the widely reported bioactive and generally tolerable range (approximately 1–20 mM) and has been shown to support cell viability and osteogenic responses in a dose‐dependent manner [39]. Meanwhile, Na^+^ levels showed no significant increase above the PBS background, suggesting no additional ionic burden under our experimental conditions (Figure S6).

Given that piezoelectric and semiconductor heterojunction systems may generate reactive oxygen species (ROS) under external stimulation [40], we further evaluated ROS production and potential mitochondrial side effects in hPDLSCs under our ultrasound parameters. Because the double‐edged sword nature of ROS, we focused on whether ultrasound induces transient ROS signaling or sustained accumulation associated with cytotoxicity [41]. DCFH‐DA staining and flow cytometry showed a detectable increase in intracellular ROS after 15 min ultrasound exposure in the POG‐HC group; however, ROS levels markedly decreased and returned close to baseline 60 min after stopping ultrasound, consistent with a transient and reversible ROS pulse rather than sustained accumulation (Figure S7). Importantly, Live/Dead staining at 24 h after stimulation revealed no significant differences in cell viability among groups, and TEM showed intact mitochondrial morphology with preserved membranes and cristae, without typical damage features such as swelling or cristae disruption (Figure S8). Notably, the inclusion of the POG‐C group enabled us to distinguish ionic microenvironment‐related effects from heterojunction‐associated safety concerns. Collectively, these data support that, under our stimulation conditions, POG‐HC did not induce significant cytotoxicity or mitochondrial ultrastructural damage.

In vivo evaluation of tissue response and biodegradability was conducted via subcutaneous implantation in rats (Figure 3F). H&E staining at various time points demonstrated progressive hydrogel degradation without evidence of fibrous encapsulation or foreign body reaction (Figure S9). Immunohistochemical staining for IL‐1β further indicated a rapid resolution of local inflammation: elevated cytokine levels observed at 1 week post‐implantation significantly decreased by the second week, nearing baseline level (Figure S10). Histological analysis of major organs showed no pathological alterations (Figure S11), supporting the systemic biocompatibility of the POG‐HC hydrogel. Collectively, these findings demonstrated that the POG‐HC piezoelectric hydrogel possesses excellent biocompatibility both in vitro and in vivo, providing a robust biosafety foundation for its application in the regenerative treatment of chronic inflammatory lesions such as periodontitis.

### 2.4 Piezoelectric Hydrogel System Promotes Osteogenic Differentiation of hPDLSCs

**FIGURE 4:**
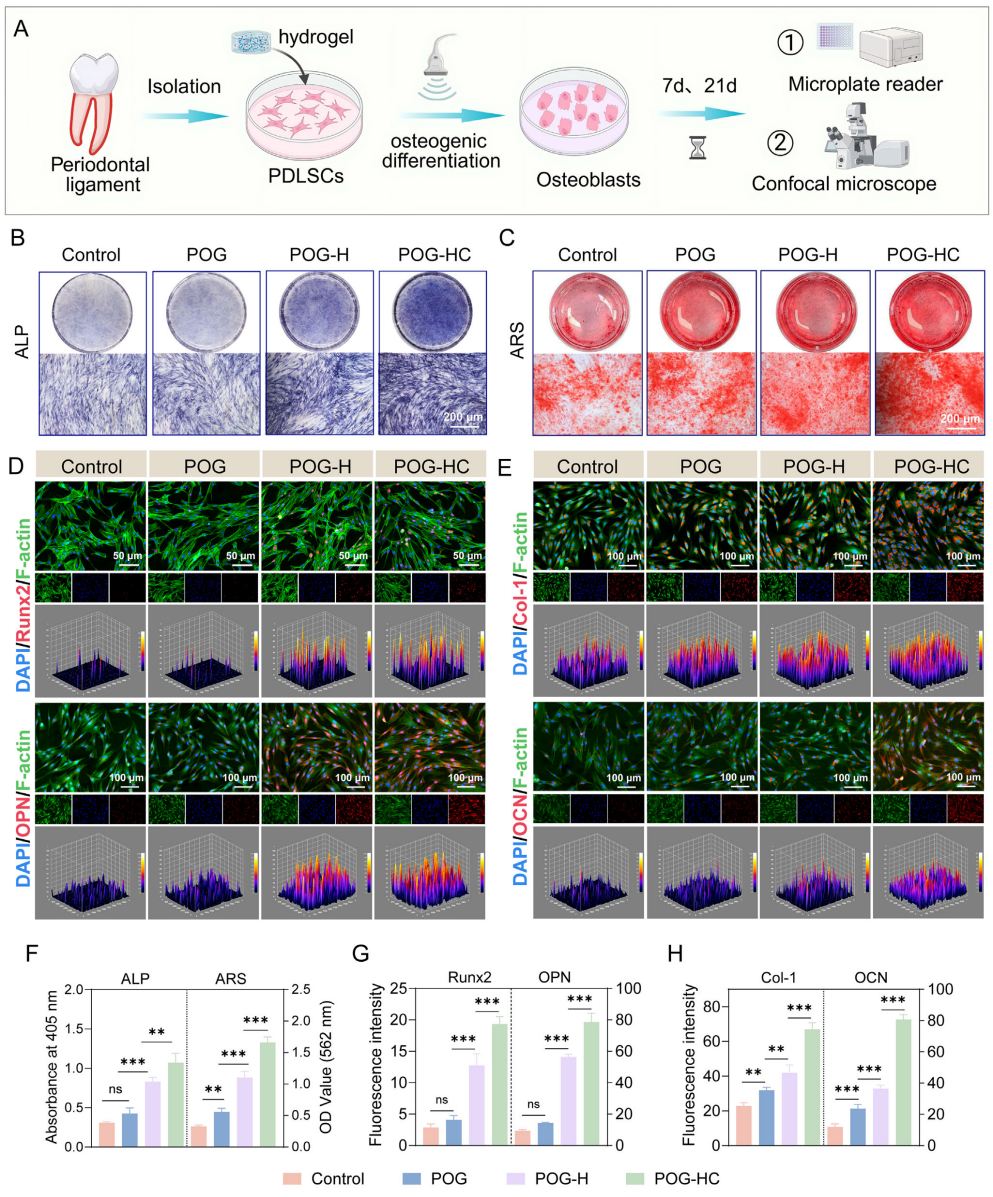
Piezoelectric hydrogel‐mediated promotion of osteogenic differentiation in hPDLSCs. (A) Schematic illustration of the osteogenic induction process following co‐culture of hPDLSCs with piezoelectric hydrogels. Created with BioRender.com. (B,C) ALP staining on day 7 and Alizarin Red S (ARS) staining on day 21 to evaluate osteogenic activity and mineral deposition of hPDLSCs (n = 4; scale bar: 200 µm). (D,E) Confocal fluorescence images showing OPN, OCN, Col‐1 (OPN/Col‐1: day 7; OCN: day 14; n = 4; scale bar: 100 µm) and Runx2 (day 3; n = 4; scale bar: 50 µm). (F) Quantitative analysis of the ALP and ARS staining results. (G,H) Quantification of fluorescence intensity for Runx2, Col‐1, OPN, and OCN expression. Data are presented as mean ± SD. ^*^ *p* < 0.05, ^**^ *p* < 0.01, ^***^ *p* < 0.001, ns, no significant.

The osteogenic differentiation of hPDLSCs is pivotal for periodontal tissue regeneration and is tightly regulated by local electrochemical cues and ionic signaling within the microenvironment [5]. In this study, we demonstrated that, even in the absence of exogenous osteoinductive proteins, the ZnO/ZnS heterojunction‐based piezoelectric hydrogel continuously generated localized electric fields upon US stimulation, thereby significantly promoting osteogenic differentiation of hPDLSCs (Figure 4A). After 7 days of culture, alkaline phosphatase (ALP) staining revealed markedly enhanced enzymatic activity in the POG‐HC group, with the absorbance at 405 nm reaching approximately 2.3‐fold that of the control group (Figure 4B,F). On day 21, Alizarin Red S staining further confirmed the mineralization potential, showing dense calcium nodule deposition in the POG‐HC group (Figure 4C,F), accompanied by significantly elevated OD values at 562 nm (*p* < 0.001), indicating robust osteoinductive activity across both early and late stages of differentiation.

To further evaluate the osteoinductive effects of each hydrogel formulation, we examined the expression of key osteogenic markers, including Runt‐related transcription factor 2 (Runx2), osteopontin (OPN), collagen type I (Col‐1), and osteocalcin (OCN). As shown in Figure 4D,E, the POG‐HC group exhibited the strongest fluorescent signals, suggesting enhanced protein secretion. 3D reconstructed images further revealed denser extracellular matrix deposition in this group. Quantitative fluorescence analysis (Figure 4G,H) showed that OPN and OCN expression levels in the POG‐HC group were approximately 8.4 and 6.7‐fold higher than those in the control, respectively, and significantly exceeded those in the POG and POG‐H groups (*p* < 0.001). Similarly, the early osteogenic transcription factor Runx2 and structural marker Col‐1 were also significantly upregulated in the POG‐HC group, with signal intensities markedly higher than in all other groups, underscoring the material's superior ability to initiate early osteogenesis.

These osteoinductive effects likely resulted from the synergistic interaction between stable piezoelectric signals and Mg^2^^+^ mediated ionic modulation under US stimulation [29]. On one hand, ZnO/ZnS heterojunctions facilitated efficient charge carrier separation, enabling the generation of sustained electric fields under mild shear stress, which may have activated mechanosensitive channels in hPDLSCs, such as voltage‐gated Ca^2^^+^ channels. On the other hand, magnesium ions reportedly upregulated Wnt/β‐catenin signaling, thereby enhancing the expression of osteogenic transcription factors such as Runx2 [42]. Therefore, the POG‐HC composite likely promotes osteogenic commitment through a coordinated mechanism involving piezoelectric activation, ionic enhancement, and signaling integration. This dual‐modality regulation not only enhanced stem cell differentiation but also provided a foundation for establishing a pro‐regenerative, immunomodulatory microenvironment in subsequent therapeutic applications.

### 2.5 Piezoelectric Hydrogel Facilitates M2 Polarization in Macrophages

**FIGURE 5:**
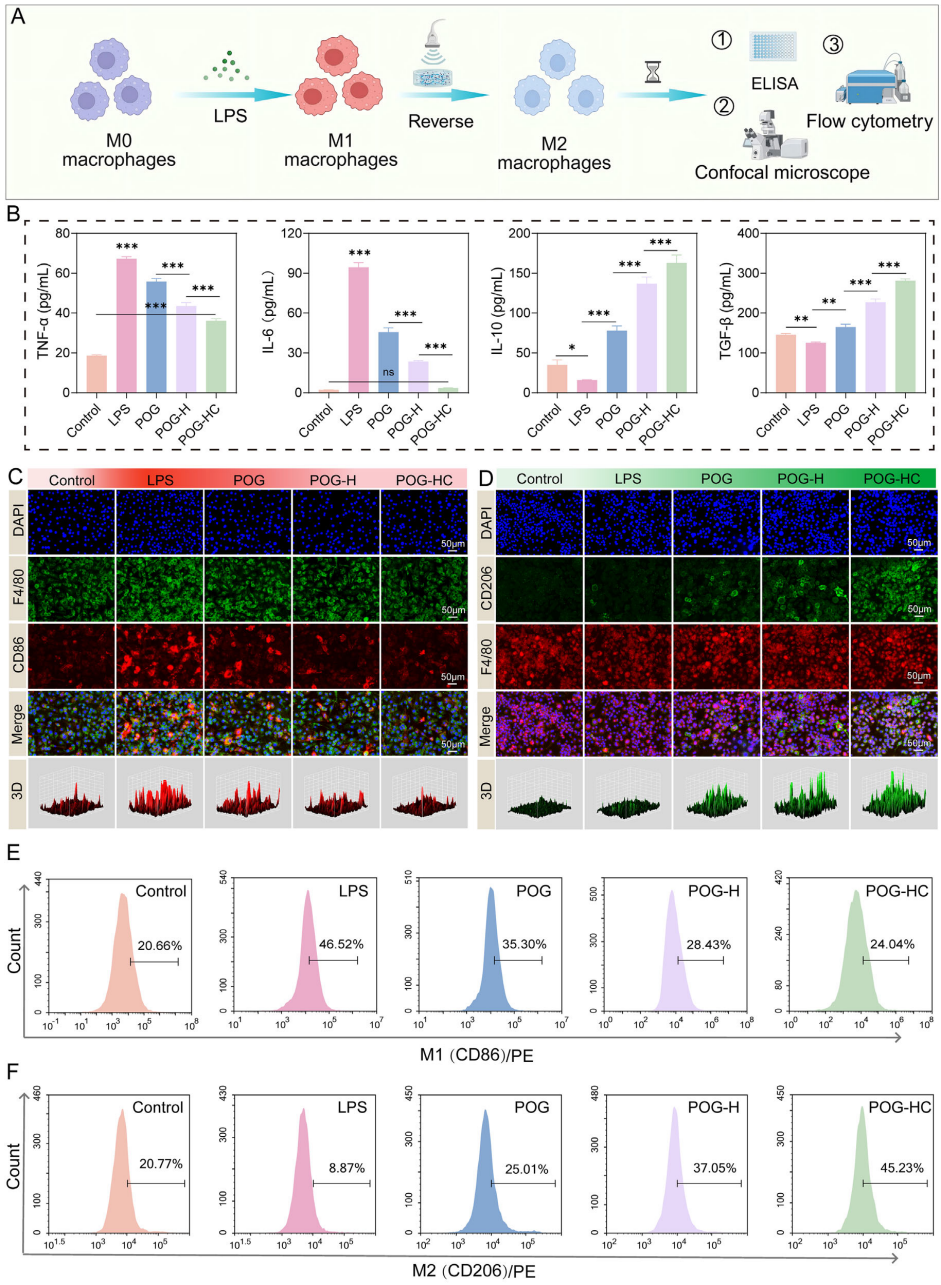
Piezoelectric hydrogel‐mediated regulation of macrophage polarization toward the M2 phenotype. (A) Schematic illustration of the experimental procedure for macrophage polarization. Created with BioRender.com. (B) ELISA analysis of inflammatory cytokines (TNF‐α, IL‐6, IL‐10, and TGF‐β) in macrophage‐conditioned media (n = 4). (C,D) Confocal fluorescence imaging of the M1 marker CD86 and the M2 marker CD206, along with a corresponding colocalization analysis (n = 4; scale bar: 50 µm). (E,F) Flow cytometric quantification of M1 (CD86^+^) and M2 (CD206^+^) macrophage populations. Data are presented as mean ± SD. ^*^ *p* < 0.05, ^**^ *p* < 0.01, ^***^ *p* < 0.001; ns: not significant.

Macrophages play a pivotal role in inflammation‐associated bone regeneration by orchestrating the immune microenvironment, and their phenotypic polarization directly influences regeneration [43]. To evaluate the immunomodulatory potential of our piezoelectric hydrogels, an LPS‐induced inflammatory model using RAW264.7 macrophages was established, and the effects of various hydrogel treatments under US stimulation on macrophage polarization and cytokine secretion were investigated (Figure 5A). As shown in Figure 5B, ELISA results revealed that both POG‐H and POG‐HC significantly suppressed the secretion of pro‐inflammatory cytokines TNF‐α and IL‐6 compared to the LPS‐only group, with POG‐HC showing the strongest inhibitory effect (*p* < 0.001). Conversely, anti‐inflammatory cytokines IL‐10 and TGF‐β were markedly upregulated in the POG‐HC group, reaching 4.7‐fold and 1.9‐fold of the control, respectively (*p* < 0.001), indicating a substantial promotion of M2 polarization.

Confocal immunofluorescence analysis further supported this finding, as enhanced expression of the M2 marker CD206 and diminished signal of the M1 marker CD86 were observed in the POG‐HC group (Figure 5C,D). Additionally, macrophages exhibited a round, elongated morphology typical of a reparative phenotype. Flow cytometric analysis quantified the polarization shift (Figure 5E,F), showing that the proportion of CD86^+^ (M1) cells decreased from 46.5% in the LPS group to 24.0% in the POG‐HC group, while CD206^+^ (M2) cells increased from 8.9% to 45.2% (*p* < 0.001), consistent with the immunofluorescence results. Collectively, these multi‐level data demonstrate that the heterojunction‐integrated and ionically conductive hydrogel, when activated by US, effectively modulates macrophage polarization by suppressing the pro‐inflammatory and promoting the anti‐inflammatory.

This immunomodulatory capability may be attributed to the hydrogel's tailored electro‐physicochemical microenvironment. On one hand, ZnO/ZnS heterojunctions generate localized piezoelectric signals under microshear, potentially activating M2 associated pathways [44]. On the other hand, magnesium ion release synergistically promotes M2 phenotype switching. In addition, the hydrogel's porous structure and high water content may help mitigate inflammatory signals and maintain a stable local environment. Compared with inert materials or single‐ion systems, the dual‐strategy design provides an effective means to modulate macrophage behavior and promote tissue repair under inflammatory conditions.

### 2.6 Piezoelectric Hydrogel Modulates the Inflammatory Microenvironment of hPDLSCs via Immune Remodeling

**FIGURE 6:**
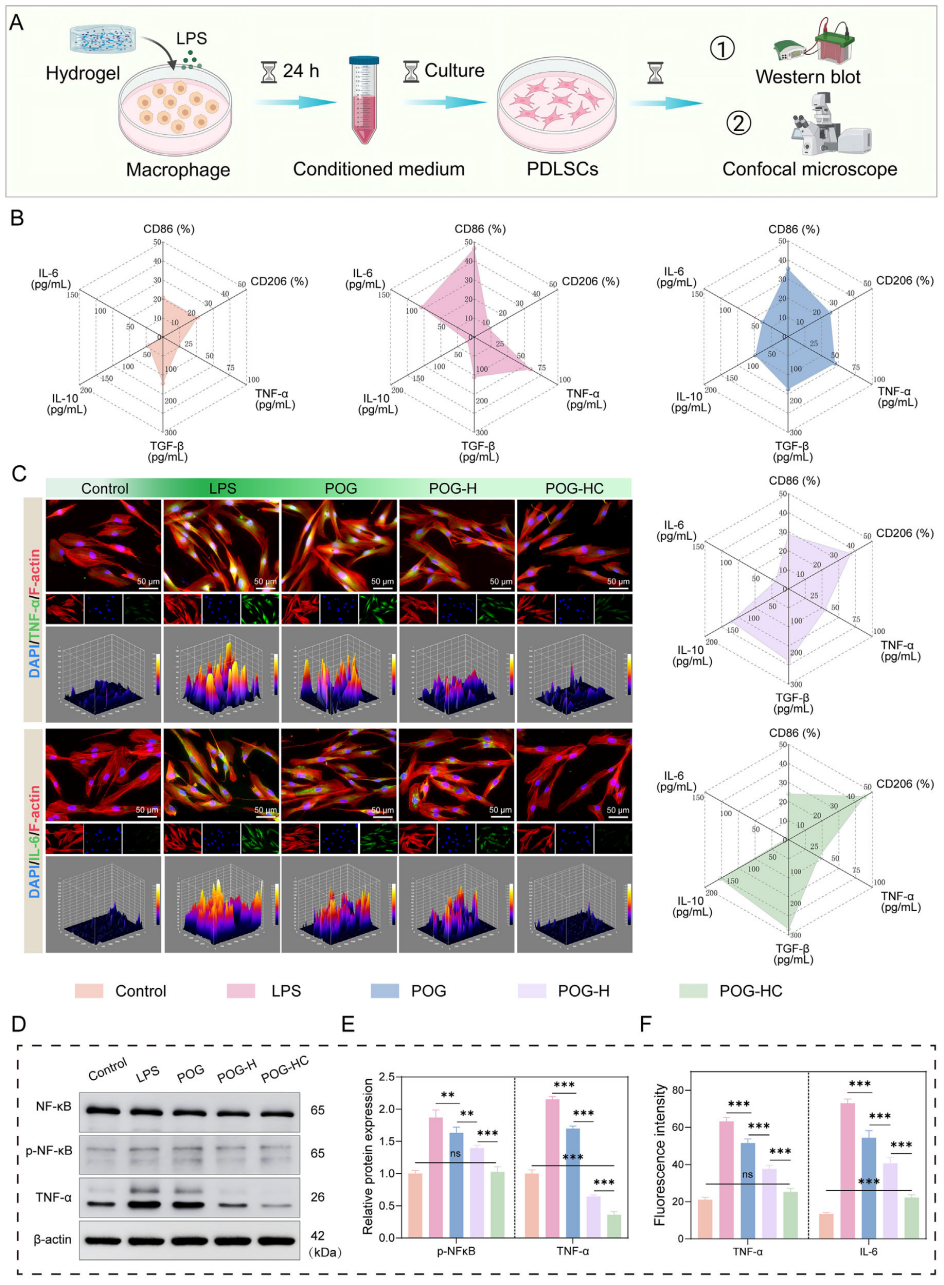
The piezoelectric hydrogel reduces hPDLSC inflammation through macrophage‐mediated immune modulation. (A) Schematic illustration of the experimental design: CM derived from hydrogel‐treated macrophages was used to regulate the inflammatory state of hPDLSCs. Created with BioRender.com. (B) Radar plot analysis of macrophage phenotype and cytokine secretion profiles. (C) Confocal fluorescence images showing the expression of inflammatory cytokines TNF‐α and IL‐6 in hPDLSCs after treatment with different CM. Green: target proteins; red: F‐actin; blue: DAPI. (D) Western blot analysis of NF‐κB pathway activation in hPDLSCs under different CM conditions. (E,F) Quantification of protein expression from Western blot and fluorescence intensity of TNF‐α and IL‐6 in PDLSCs. Data are presented as mean ± SD; ^*^ *p* < 0.05, ^**^ *p* < 0.01, ^***^ *p* < 0.001; ns: not significant.

During inflammatory tissue repair, macrophages and mesenchymal stem cells dynamically interact through paracrine signaling, which critically governs immune homeostasis within the regenerative niche and ultimately influences healing outcomes. Proinflammatory cytokines secreted by activated M1 macrophages, such as TNF‐α and IL‐6, impair the proliferation and differentiation of hPDLSCs, whereas polarization toward an M2 phenotype helps alleviate inflammation and restore stem cell function [45]. The radar plot summarizing macrophage polarization (Figure 6B) showed that POG‐HC markedly reduced the expression of CD86, TNF‐α, and IL‐6, while upregulating CD206, IL‐10, and TGF‐β, indicating a favorable immunomodulatory profile.

To further determine whether this piezoelectric hydrogel system could regulate the osteogenic potential of periodontal ligament stem cells and facilitate inflammatory resolution under an inflammatory microenvironment [46, 47], we established a classical macrophage–stem cell conditioned medium (CM) crosstalk model (Figure 6A). Specifically, RAW264.7 macrophages were first stimulated with LPS to induce an inflammatory state, then co‐incubated with different hydrogels; the supernatants were subsequently collected as CM and applied to hPDLSCs. We then evaluated the inflammatory status of hPDLSCs exposed to the different CM preparations. Confocal imaging showed that TNF‐α and IL‐6 expression was strongly upregulated in the LPS group, whereas it was markedly reduced in the POG‐HC group, indicating an alleviated inflammatory state (Figure 6C). Quantitative analysis further revealed that the fluorescence intensities of TNF‐α and IL‐6 in the POG‐HC group decreased to approximately 39.9% and 30.5% of those in the LPS group, respectively (*p* < 0.001; Figure 6F). Moreover, ALP and ARS staining demonstrated that CM derived from LPS‐activated macrophages markedly suppressed ALP activity and mineralized nodule formation in hPDLSCs, whereas CM from macrophages treated with POG‐H or POG‐HC partially restored ALP staining and rescued mineral deposition (Figure S12). Collectively, these results indicate that POG‐HC can remodel the inflammatory niche through immunomodulation, thereby improving the inflammatory phenotype of hPDLSCs and promoting their osteogenic differentiation under inflammatory conditions.

The NF‐κB signaling pathway is known to link macrophage polarization with the inflammatory responses of hPDLSCs. In inflammatory environments, this pathway not only skews macrophages toward the M1 phenotype but also promotes the release of proinflammatory cytokines such as TNF‐α and IL‐6, thereby suppressing the regenerative potential of hPDLSCs [48]. Therefore, we examined NF‐κB pathway activation in hPDLSCs to elucidate the mechanism underlying the anti‐inflammatory effects of our hydrogel system (Figure 6D). Western blot analysis revealed that LPS stimulation induced robust upregulation of p‐NF‐κB and TNF‐α, while POG‐HC treatment significantly downregulated both proteins (*p* < 0.001; Figure 6E). These findings were consistent with the observed cytokine trends and further confirmed that the POG‐HC hydrogel system established a more immune‐permissive environment in vitro.

### 2.7 Therapeutic Efficacy of Piezoelectric Hydrogel in a Rat Model of Periodontitis

**FIGURE 7:**
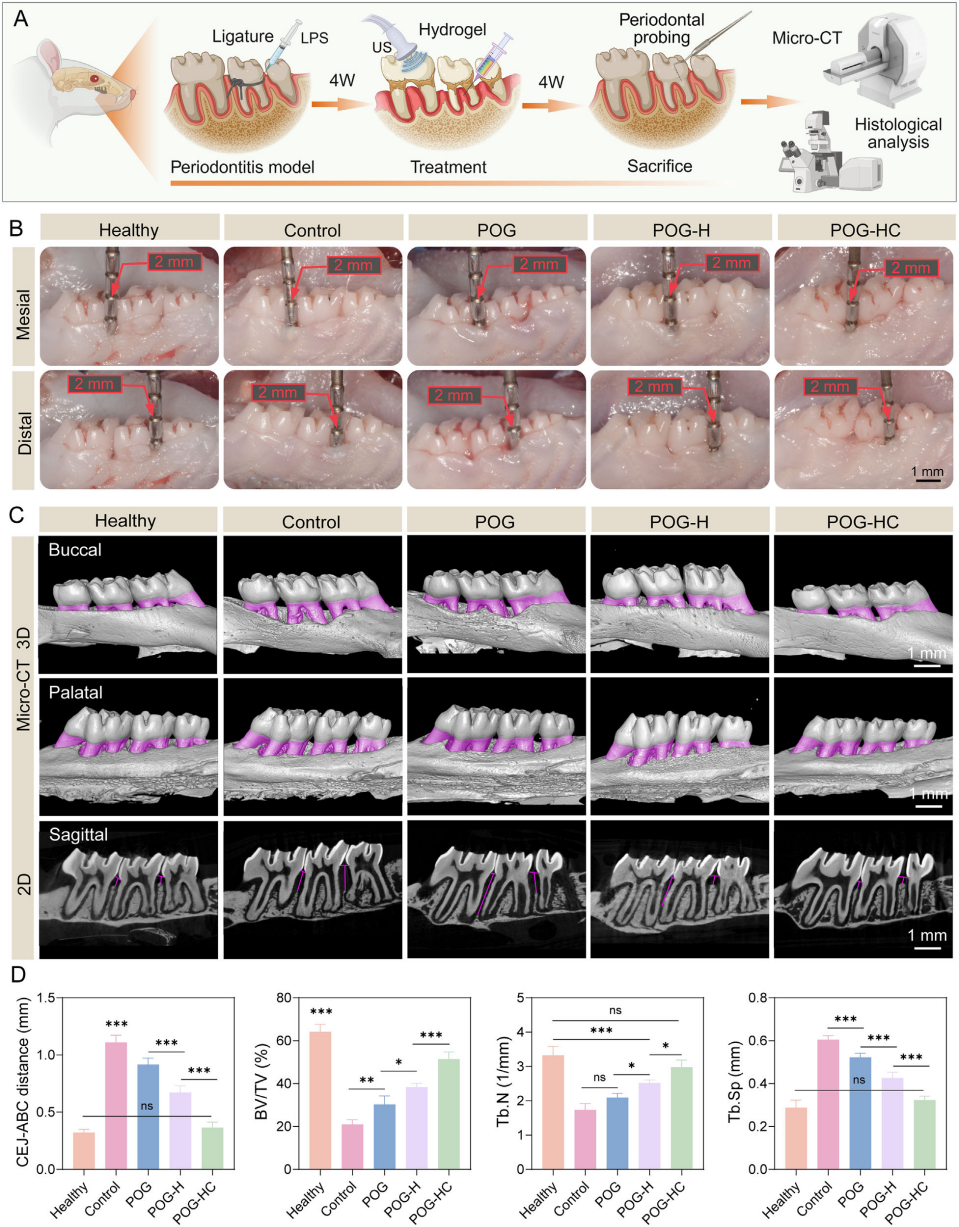
Therapeutic effects of the piezoelectric hydrogel system in a rat periodontitis model. (A) Schematic illustration of the periodontitis model established by ligature placement and LPS injection, followed by piezoelectric hydrogel treatment. Created with BioRender.com. (B) Periodontal probing performed at the mesial and distal sites of the palatal aspect of the maxillary second molars in rats (n = 4; scale bar: 1 mm). (C) Micro‐CT 3D reconstructions (buccal and palatal views) and sagittal cross‐sectional images showing alveolar bone regeneration (scale bar: 1 mm). (D) Quantitative analysis of the distance between CEJ–ABC, BV/TV, Tb.N, and Tb.Sp. Data are presented as mean ± SD. ^*^ *p* < 0.05, ^**^ *p* < 0.01, ^***^ *p* < 0.001, ns, no significant.

To evaluate the regenerative performance of the composite piezoelectric hydrogel under complex inflammatory conditions, a chronic periodontitis model was established in rats. As shown in Figure 7A, different hydrogel formulations were locally injected into the periodontal defect sites, and therapeutic outcomes were assessed after four weeks of treatment. Periodontal probing (Figure 7B) revealed that the Control group exhibited deep periodontal pockets, with mesial and distal probing depths > 1 mm, indicating severe structural damage. In contrast, the POG‐HC group displayed tight gingival attachment with significantly reduced probing depths, suggesting effective reconstruction of periodontal tissue. Micro‐CT analysis further confirmed the extent of alveolar bone regeneration. As shown in Figure 7C, extensive buccal and palatal bone resorption was observed in the Control and POG groups, while the POG‐HC group showed continuous and well‐integrated bone formation, with alveolar crest morphology resembling that of healthy controls. Sagittal sections demonstrated that dense and homogeneous trabecular bone had formed between the roots in the POG‐HC group.

Quantitative metrics (Figure 7D) showed that the CEJ–ABC distance in the POG‐HC group decreased significantly to 0.36 ± 0.05 mm compared to 1.11 ± 0.03 mm in the Control group (*p* < 0.001), indicating substantial improvement in periodontal attachment. Moreover, the bone volume fraction (BV/TV) increased to 51.2%, approximately 30% higher than in the Control group. The trabecular number (Tb.N) also increased, while the trabecular separation (Tb.Sp) decreased, collectively reflecting improved bone quality in the POG‐HC group relative to Control, POG, and POG‐H groups (*p* < 0.05). These findings highlight the excellent tissue integration and bone‐regenerative capacity of the piezoelectric hydrogel in an inflammatory microenvironment.

### 2.8 Piezoelectric Hydrogel Promotes Osteoimmune Bone Regeneration

**FIGURE 8:**
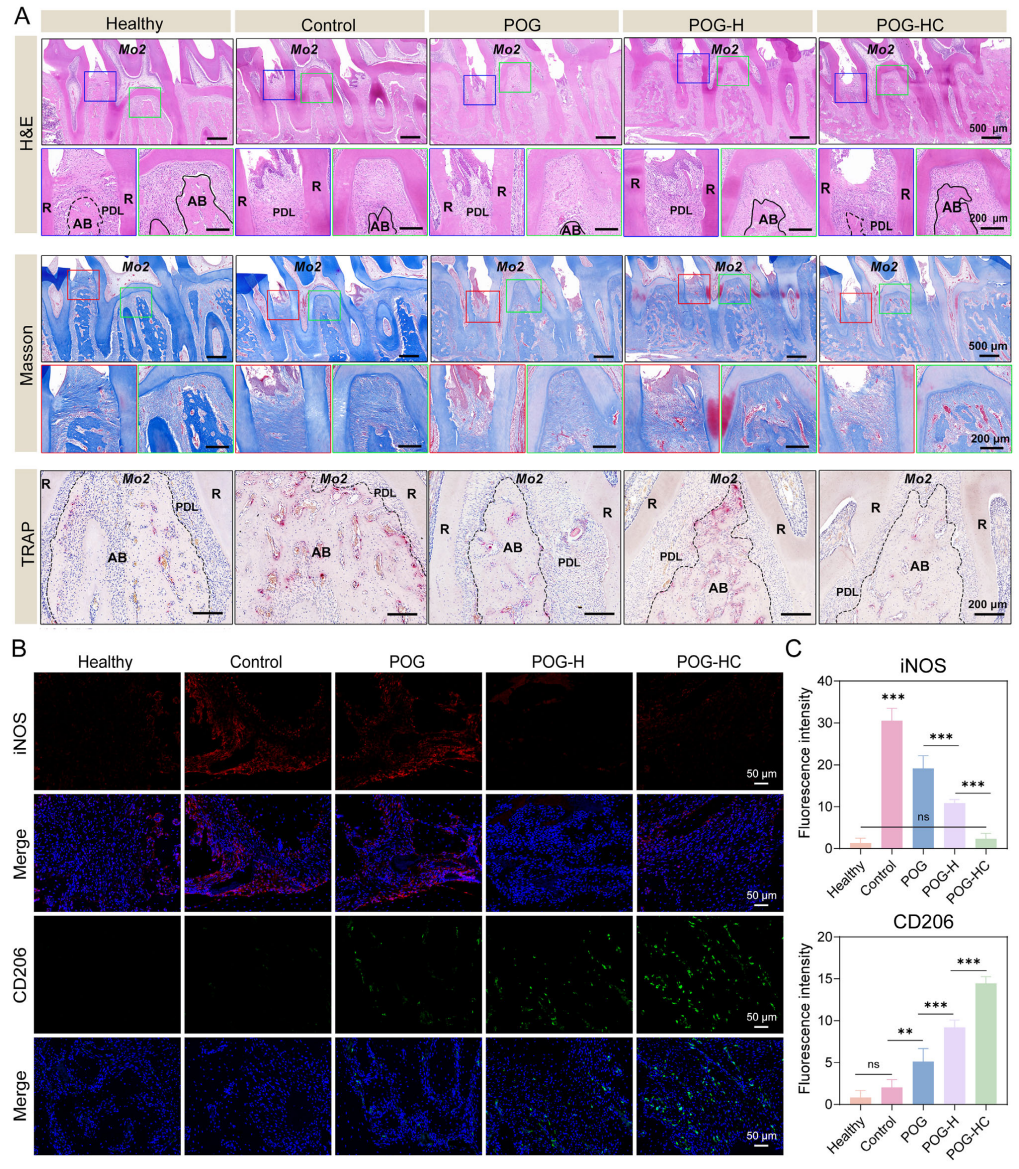
Histological analysis of piezoelectric hydrogel–mediated alveolar bone regeneration. (A) H&E and Masson's trichrome staining of the alveolar bone and periodontal ligament (PDL) in different treatment groups (AB, alveolar bone; R, root; *Mo2*, second molar). In TRAP staining, red signals indicate regions with active osteoclasts (n = 4; scale bars: 500 µm/200 µm). (B) Immunofluorescence of M1 macrophage marker (iNOS) and M2 marker (CD206) in inflamed regions, with DAPI nuclear counterstain (n = 4; scale bar: 50 µm). (C) Quantification of iNOS and CD206 fluorescence intensities to assess macrophage polarization under different treatments. Data shown as mean ± SD; ^*^ *p* < 0.05, ^**^ *p* < 0.01, ^***^ *p* < 0.001; ns, not significant.

Effective reconstruction of the alveolar bone requires both osteogenesis and immune homeostasis within an inflammatory microenvironment. To delineate the in vivo inflammation–repair coupling, H&E, Masson, and TRAP staining were performed to assess tissue architecture, collagen fiber organization, and osteoclast activity across groups (Figure 8A). H&E/Masson staining revealed intact periodontal ligament (PDL), dense alveolar bone, and well‐aligned fibers in the Healthy group at the second molar (*Mo2*) region, whereas the Control group exhibited pronounced inflammatory destruction with disorganized PDL and irregular bone resorption. In contrast, the POG‐HC group showed well‐restored PDL architecture, densely packed fiber bundles, and a well‐defined alveolar crest, outperforming POG and POG‐H. TRAP staining further demonstrated abundant TRAP^+^ osteoclasts along the bone surface in the Control group, while the TRAP^+^ area was markedly reduced in the POG‐HC group, indicating effective suppression of inflammation‐driven bone resorption.

To assess immune modulation, macrophage markers iNOS/CD206 were examined (Figure 8B). Immunofluorescence showed strong iNOS and weak CD206 in the Control group, while the POG‐HC group showed decreased iNOS and increased CD206, suggesting a shift toward M2 macrophage predominance. Quantitative analysis confirmed this phenotypic shift (Figure 8C, *p* < 0.001). Collectively, the POG‐HC piezoelectric hydrogel suppresses osteoclast activity while inducing macrophage M2 polarization within the periodontitis microenvironment, thereby coordinating bone regeneration with immune remodeling and highlighting its engineered, bioadaptive regenerative potential.

### 2.9 Transcriptomics Reveal the Mechanism of POG‐HC Piezoelectric Hydrogel in Periodontitis

**FIGURE 9:**
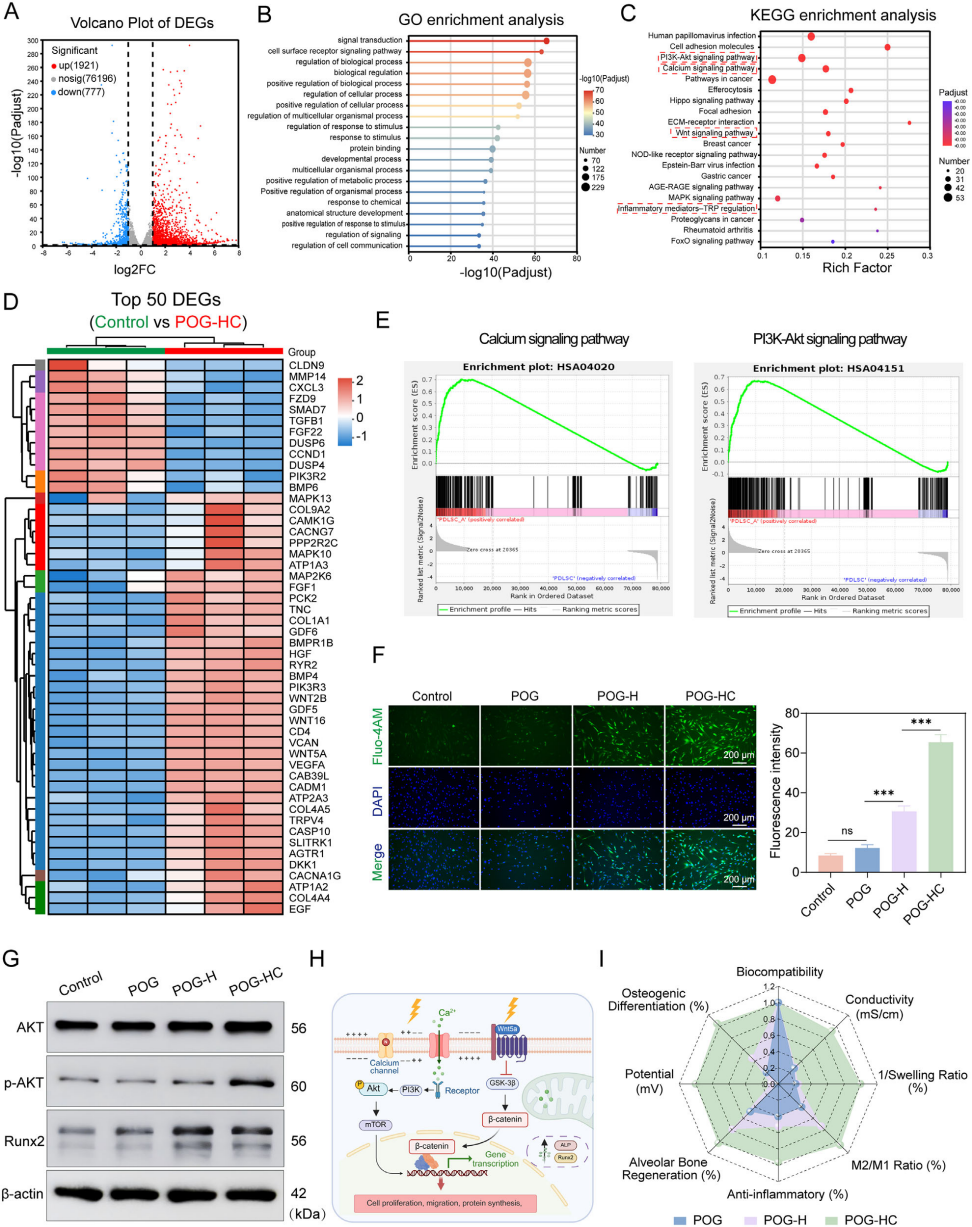
Molecular mechanisms of POG‐HC piezoelectric hydrogel therapy for periodontitis. (A) Volcano plot of differentially expressed genes (DEGs) from RNA‐seq (red, upregulated; blue, downregulated). (B) GO enrichment analysis reveals significantly enriched biological processes. (C) KEGG pathway enrichment showing significant activation of osteogenesis‐related pathways, including calcium signaling, PI3K/Akt, and adherens junctions. (D) Heatmap of DEGs associated with osteogenesis and signal transduction. (E) Gene Set Enrichment Analysis (GSEA) confirms significant enrichment of calcium signaling and PI3K/Akt pathways. (F) Intracellular Ca^2^^+^ imaging by Fluo‐4 AM (n = 3; scale bar: 200 µm). (G) Western blot analysis of proteins linked to osteogenesis and signaling pathways. (H) Proposed mechanism by which the piezoelectric hydrogel promotes osteogenesis‐related signaling (Created with BioRender.com). (I) Radar chart summarizing the comprehensive performance of the POG‐HC piezoelectric hydrogel.

To elucidate how the POG‐HC piezoelectric hydrogel regulates hPDLSCs, we performed transcriptome sequencing on Control and POG‐HC groups. The volcano plot identified 2,698 significantly differentially expressed genes (DEGs) in the POG‐HC group, with 1,921 upregulated and 777 downregulated, indicating broad transcriptional reprogramming (Figure 9A; Figure S13). The heatmap of the top 50 DEGs further showed marked upregulation of multiple regulatory factors after POG‐HC treatment (e.g., NFATC2, P2X receptors, TRP channels, MAPK), suggesting enhanced electromechanical coupling between the material and cells (Figure 9D). Meanwhile, key genes in canonical osteogenic pathways—including PIK3R3, WNT5A, BMP4, and COL4A—were concurrently upregulated. GO and KEGG enrichment analyses confirmed that these DEGs were significantly enriched in several osteogenesis‐related signaling axes (Figure 9B,C), notably Ca^2^^+^ signaling, PI3K/AKT, and Wnt/β‐catenin, forming a multi‐nodal regulatory network (Figure 9E; Figure S14).

To validate the transcriptomic findings, intracellular Ca^2^^+^ was labeled with the fluorescent probe Fluo‐4 AM, and Ca^2^^+^ dynamics in hPDLSCs were visualized by confocal microscopy (Figure 9F). The POG‐HC group displayed markedly increased green fluorescence intensity, indicating elevated intracellular Ca^2^^+^ and confirming that the material activates mechanosensitive and electrosensitive channels to induce second‐messenger Ca^2^^+^ influx as a key node. Western blotting further verified pathway activation: p‐AKT was significantly elevated with an increased p‐AKT/AKT ratio, indicating sustained activation of the PI3K/AKT pathway. Concurrently, β‐catenin and osteogenic markers Runx2 and ALP were upregulated upon POG‐HC treatment (Figure 9G; Figure S15). To further validate the key roles of Ca^2+^ and PI3K/AKT signaling in this process and to probe their causal relationships, we added inhibitor intervention groups and a salt‐only control group (Figure S16). Specifically, we introduced a salt control hydrogel, POG‐C, which possesses an ionically conductive network but lacks the ZnO/ZnS piezoelectric heterojunction, to distinguish the contribution of the ionic microenvironment from that of the piezoelectric heterojunction. In parallel, we applied the intracellular Ca^2+^ chelator BAPTA‐AM and the PI3K inhibitor LY294002 for pathway perturbation. Flow cytometry showed that intracellular Ca^2+^ fluorescence was markedly increased under POG‐HC conditions, whereas the POG‐C group exhibited only a mild increase compared with the untreated control; after BAPTA‐AM treatment, the Ca^2+^ signal decreased to near baseline levels. Consistently, Western blotting demonstrated that BAPTA‐AM markedly reduced p‐AKT as well as the osteogenic markers Runx2 and ALP, yet these levels did not fully return to those of the control group. These findings indicate that Ca^2+^ influx is an important contributing node for downstream osteogenic activation, but it is unlikely to be the sole convergence point, and the piezoelectric and ionic microenvironments may also act through additional parallel pathways. Furthermore, under POG‐HC + LY294002 conditions, p‐AKT was effectively suppressed, with concomitant downregulation of Runx2 and ALP, whereas the overall intracellular Ca^2+^ fluorescence remained largely unchanged. This supports PI3K/AKT as a key downstream axis for osteogenic signaling in our system, and under our experimental conditions, we did not observe an apparent feedback effect of PI3K/AKT on Ca^2+^ influx.

Integrating RNA‐seq with experimental validation, we propose an osteogenic regulatory model in which piezoelectric signaling activates Ca^2^^+^ channels and co‐activates PI3K/AKT and Wnt/β‐catenin pathways (Figure 9H). In addition, multidimensional comparisons demonstrated that POG‐HC outperformed POG and POG‐H in biocompatibility, anti‐inflammatory capacity, osteogenic performance, immunomodulation, and in vivo bone regeneration (Figure 9I), underscoring its engineered advantage in synergistic performance. Collectively, the ZnO/ZnS heterojunction piezoelectric hydrogel, under US stimulation, persistently delivers micro‐electric fields that enhance Ca^2^^+^ signaling, cooperatively activate multiple osteogenic pathways, reprogram hPDLSCs phenotypes, and promote osteogenic differentiation.

## 3 Conclusions

By integrating a *two‐pronged approach* and a *one‐stone‐multi‐birds* strategy, we engineered a ZnO/ZnS heterojunction enabled piezoelectric hydrogel with enhanced piezoelectric response, high conductivity, and microenvironmental adaptability, thereby addressing key limitations of conventional piezoelectric materials in periodontitis therapy. The ZnO/ZnS heterojunction, augmented by interfacial and defect engineering, markedly enhanced charge separation and piezoelectric output; the Mg^2^^+^/citrate dual‐salt system endowed high ionic conductivity, mechanical robustness, and pro‐osteogenic activity; and the multinetwork hydrogel ensured injectability and long‐term stability. As a result, the platform synergistically induced osteogenic differentiation of hPDLSCs and M2 polarization of macrophages in vitro, and significantly promoted periodontal tissue repair and bone regeneration in vivo. Mechanistically, these effects were associated with activation of the PI3K/AKT signaling pathway, accompanied by crosstalk with the Wnt/β‐catenin cascade, facilitating remodeling of the piezoelectric–bio interface. This innovative material system offers a broadly applicable paradigm for the treatment of infectious bone defects, metabolic bone disorders, and arthritis, further advancing the clinical translation of piezoelectric bioelectric strategies in regenerative medicine.

## 4 Experimental Section

Experimental details are available in the Supporting Information. hPDLSCs and RAW264.7 cells were obtained from commercial and institutional sources, respectively, and were routinely tested to be mycoplasma‐free (Supporting Information for details). The sensing performance of the hydrogels on the volunteer's body motions was conducted with the consent of the volunteer. All experimental animals were provided by Shanghai Shengchang Biotechnology Co., Ltd., and all animal experiments were approved by the Animal Ethics and Use Committee of Shanghai Shengchang Biotechnology Co., Ltd. (Ethics No. 2025‐01‐KOYY‐WXL‐126).

### 4.1 Statistical Analysis

Statistical analysis was conducted by using GraphPad Prism 8 statistical software. Differences between groups were analyzed by one‐way analysis of variance (ANOVA), followed by Tukey's multiple comparison tests. Data represent the mean ± SD of at least three replicates. ^*^ *p* < 0.05, ^**^ *p* < 0.01, and ^***^ *p* < 0.001 were regarded as statistically significant. Additionally, “ns” denoted no significant difference.

## Author Contributions

X.W., R.Z., S.F., and Q.Z. conceived and designed the project. B.L., H.X., X.S., S.Y., and X.W. put forward many constructive suggestions and helped with the implementation of the project. Q.Z., C.L., S.Y., and S.F. performed all data collection. M.Z. and C.H. conducted the analysis of experimental data. Q.Z. and S.F. wrote the manuscript. X.W., R.Z., S.F., X.S., and Q.Z. corrected the manuscript. Others carried out auxiliary work related to these experiments.

## Conflicts of Interest

The authors declare no conflicts of interest.

## Supporting information




**Supporting file**: advs74356‐sup‐0001‐SuppMat.docx.

## Data Availability

The data that support the findings of this study are available from the corresponding author upon reasonable request.
