# Supplementary material for: Dual‐Strategy Design of Heterojunction‐Enhanced Piezoelectric Hydrogels for Periodontitis Treatment
Source: Adv Sci (Weinh). 2026 Feb 13;13(22):e00017. doi: 10.1002/advs.202600017 (PMC13088289; doi:10.1002/advs.202600017)
Supplement: Supplementary file 1 — Supporting file: advs74356‐sup‐0001‐SuppMat.docx. [file ADVS-13-e00017-s001.docx]

**Supporting Information for**

### Dual-Strategy Design of Heterojunction-Enhanced Piezoelectric Hydrogels for Periodontitis Treatment

*Qiangqiang Zhou^1,3†^, Shaojun Fang^2†^, Changyi Li^1,3†^, Mengqi Zhou^1,3^, Xin Sui ^1,3^, Chen Hu^1,3^, Huaxing Xu^1,3^, Shiyi Yang^2^, Bingqiang Lu^1,3^, Rongjun Zhang^2*^, Xiaoling Wei^1,3*^*

*^1^ Department of Endodontics, Shanghai Stomatological Hospital and School of Stomatology, Fudan University, Shanghai, 200001, China.*

*^2^* *Key Laboratory of Micro and Nano Photonic Structures (MOE), Shanghai Engineering Research Center of Ultra-Precision Optical Manufacturing, College of Future Information Technology, Fudan University, Shanghai 200433, China.*

*^3^ Shanghai Key Laboratory of Craniomaxillofacial Development and Diseases, Fudan University, Shanghai, 200001, China.*

**Corresponding authors. E-mail: rjzhang@fudan.edu.cn; xiaoling_wei@fudan.edu.cn.*

*^†^These authors contributed equally to this work.*

**This file includes:**

**Results: Figure S1-S16.**

**Materials and Methods.**


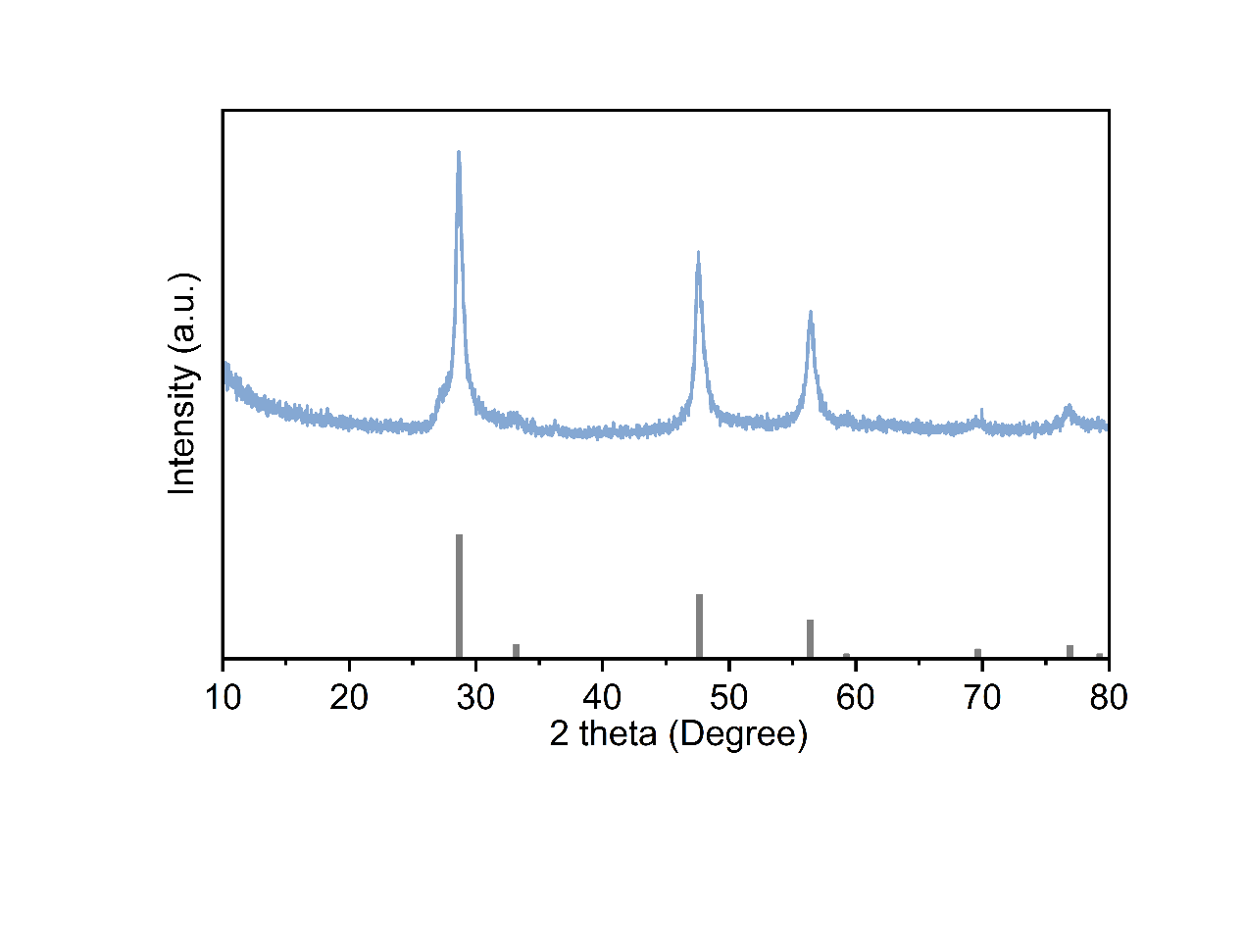


**Figure S1.** XRD patterns of ZnO/ZnS_-0.08_ compared to the standard XRD card for ZnS (PDF: #05-0566).


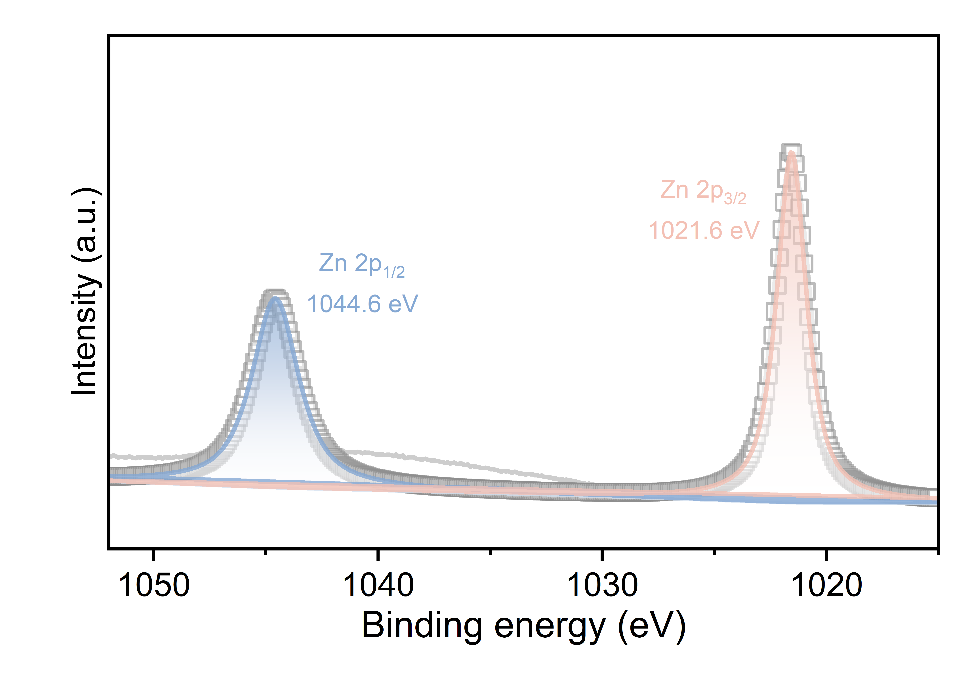


**Figure S2.** Zn 2p spectra of ZnO/ZnS_-0.08_.


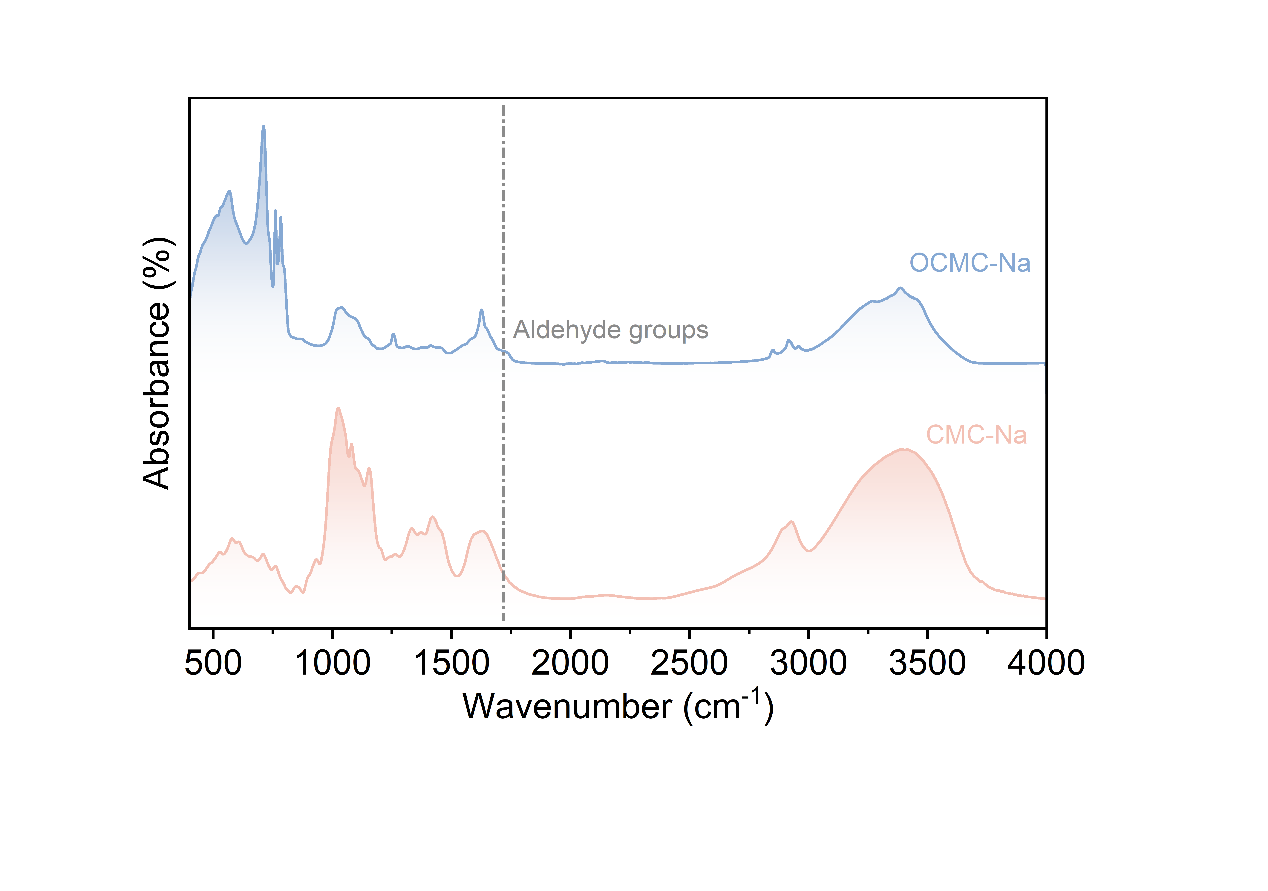


**Figure S3.** FTIR spectra of OCMC-Na and CMC-Na.

Compared to CMC-Na, OCMC-Na exhibits a slight peak around 1700 cm^-1^ corresponding to the aldehyde groups, indicating partial oxidation of carboxyl groups to aldehydes in the structure.


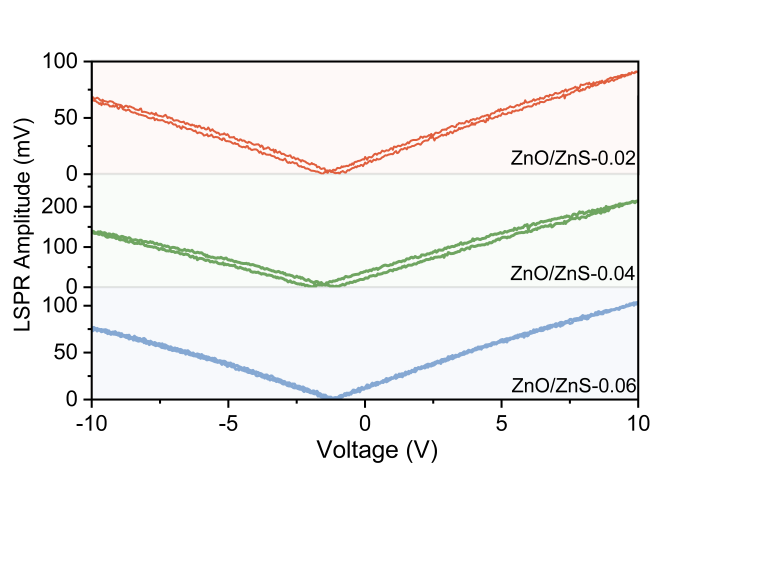


**Figure S4.** PFM amplitude–voltage loops of ZnO/ZnS heterojunctions with different ZnS ratios.


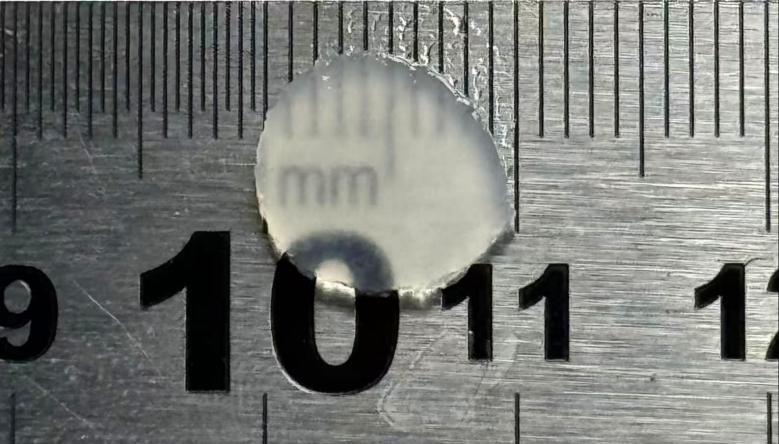


**Figure S5.** Digital photograph of the high-transparency POG-HC hydrogel.


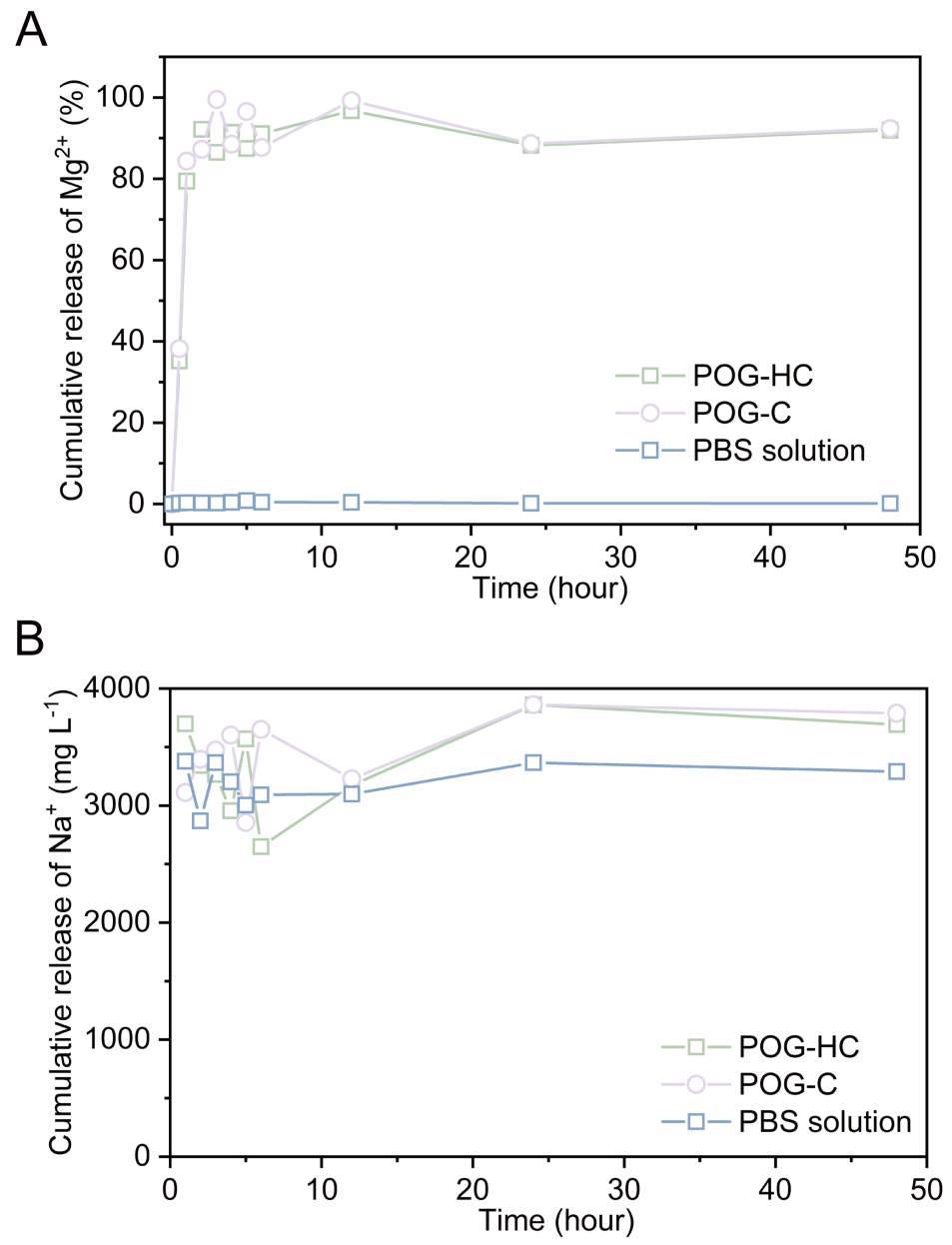


**Figure S6.** Cumulative release profiles of Mg²⁺ and Na⁺ ions from the piezoelectric hydrogels. A) Cumulative Mg²⁺ release. B) Cumulative Na⁺ release. Release was measured for POG-HC and POG-C hydrogels (dual-salt network without the ZnO/ZnS heterojunction), with PBS solution used as a blank control.


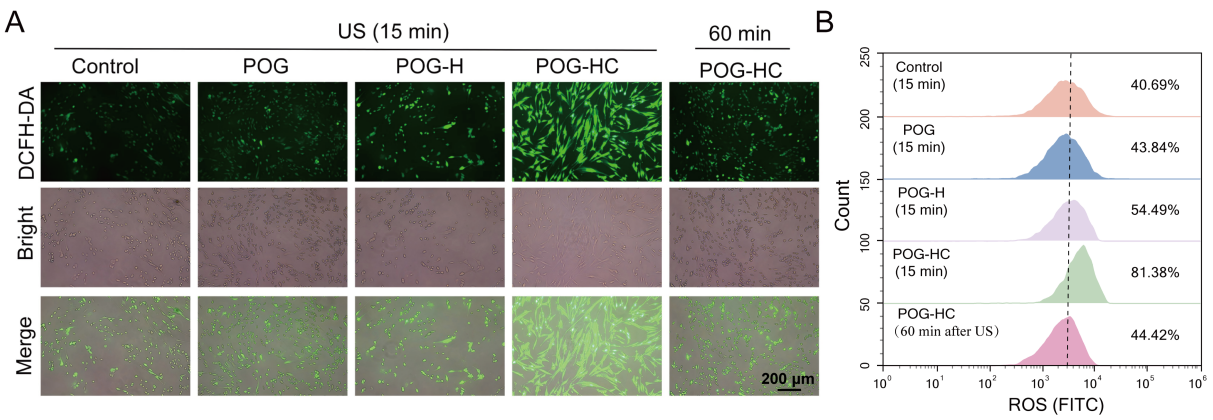


**Figure S7. Intracellular ROS levels in PDLSCs cultured with different hydrogels under ultrasound stimulation.** A) Representative DCFH-DA fluorescence images (green) after 15 min ultrasound exposure for Control, POG, POG-H, and POG-HC, and after an additional 60 min recovery period for the POG-HC group. Brightfield and merged images are shown. Scale bar: 200 μm. B) Flow cytometry quantification of fluorescence intensity (FITC channel) corresponding to intracellular ROS levels for each group.


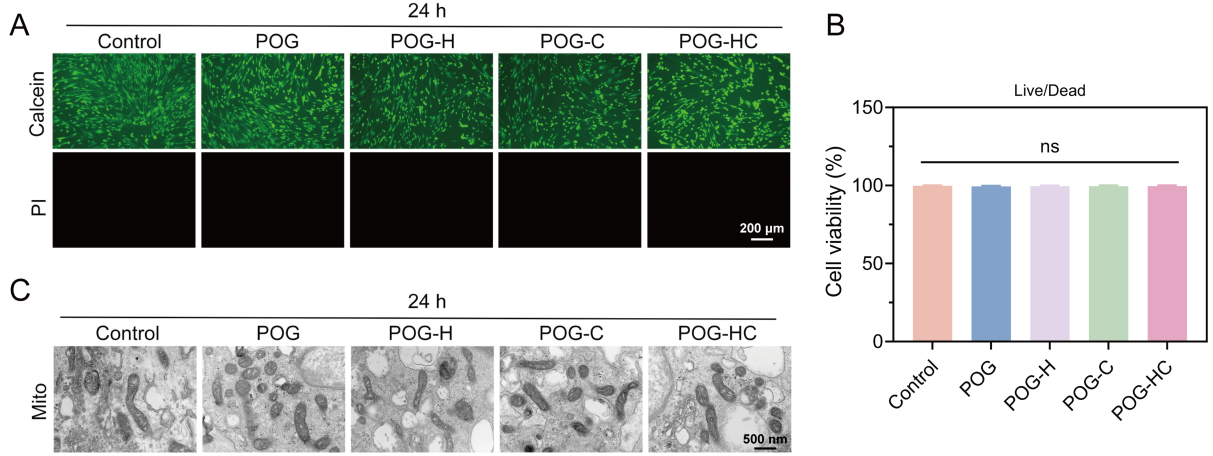


**Figure S8. Effects of ultrasound stimulation on cell viability and mitochondrial ultrastructure in different hydrogel groups.** A) Representative live/dead staining images of cells cultured for 24 h after 15 min ultrasound exposure (Calcein-AM, green, live cells; PI, red, dead cells). Scale bar: 200 μm. B) Quantification of cell viability based on live/dead staining, showing no significant differences among groups (ns). C) Representative transmission electron microscopy (TEM) images of mitochondria acquired at 24 h after 15 min ultrasound exposure. Scale bar: 500 nm.


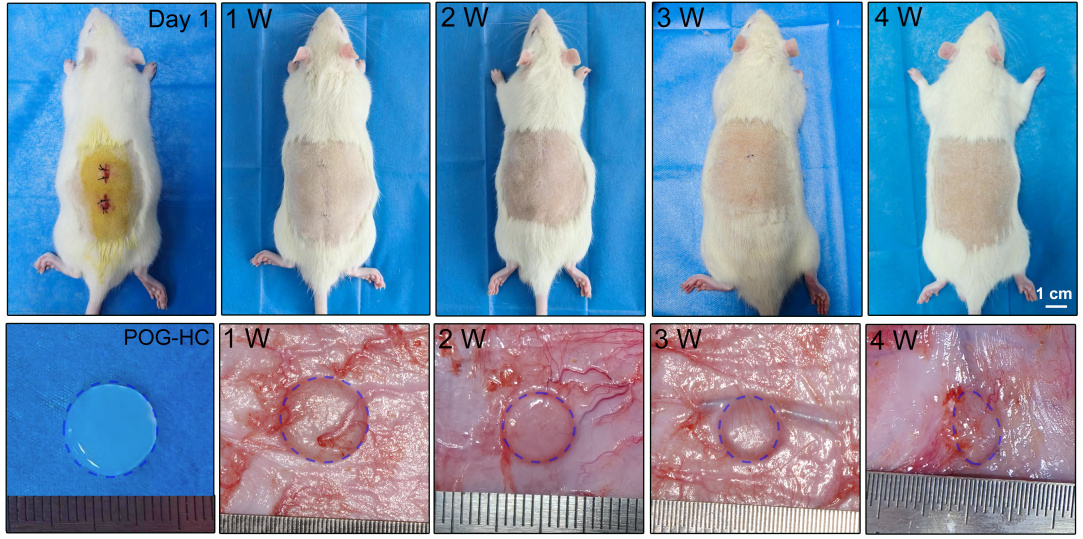


**Figure S9.** Digital images of subcutaneous implantation and macroscopic appearance of the POG-HC hydrogel.


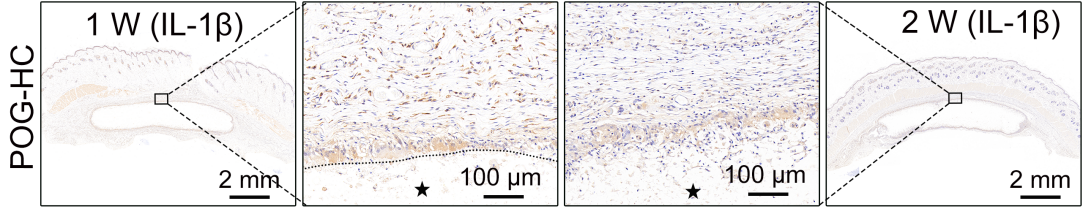


**Figure S10.** Immunohistochemical staining of IL-1β in subcutaneous tissues at 1 and 2 weeks post-implantation of the POG-HC hydrogel.


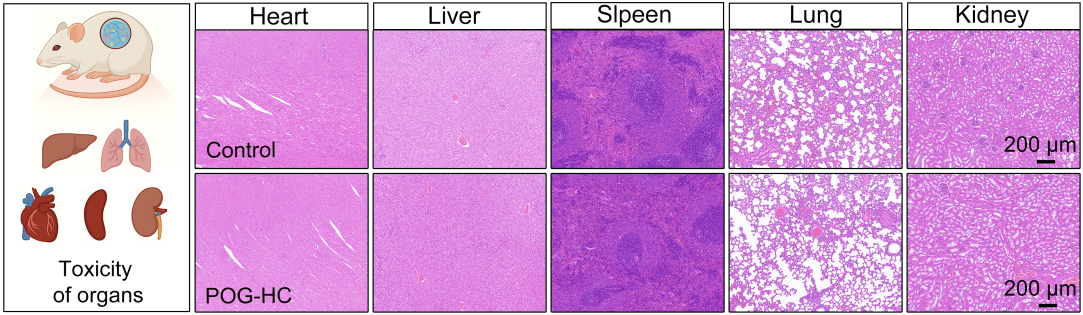


**Figure S11.** Histological analysis of major organs (heart, liver, spleen, lungs, and kidneys) with the POG-HC hydrogel.


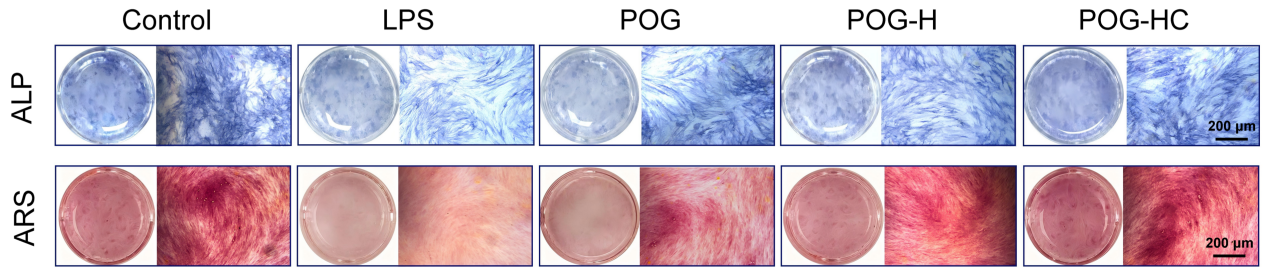


**Figure S12.** ALP and ARS staining of hPDLSCs cultured with conditioned medium collected from LPS-stimulated macrophages pretreated with different hydrogels.


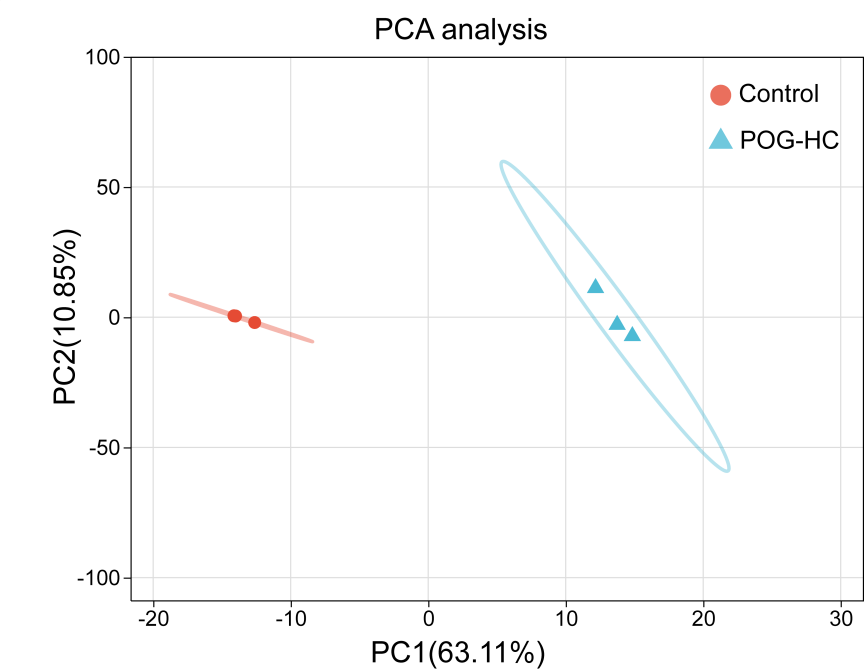


**Figure S13.** PCA plot of sample comparisons.


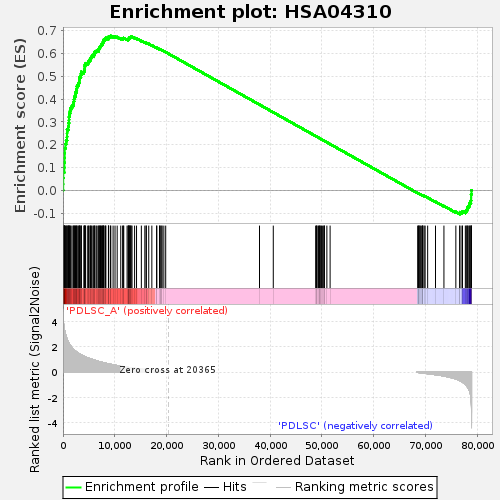


**Figure S14.** GSEA analysis of the Wnt signaling pathway.


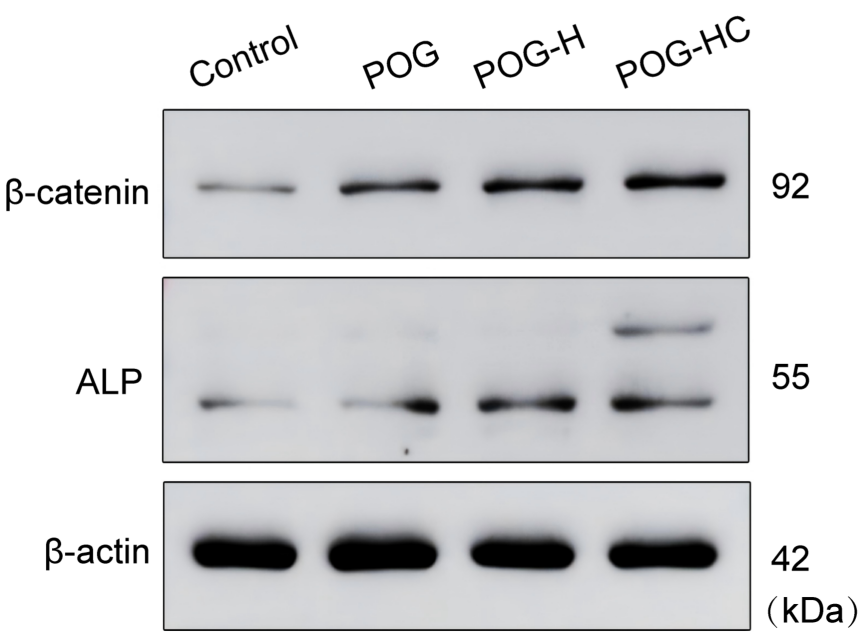


**Figure S15.** Western blot analysis of the relative expression levels of β-catenin and ALP proteins.


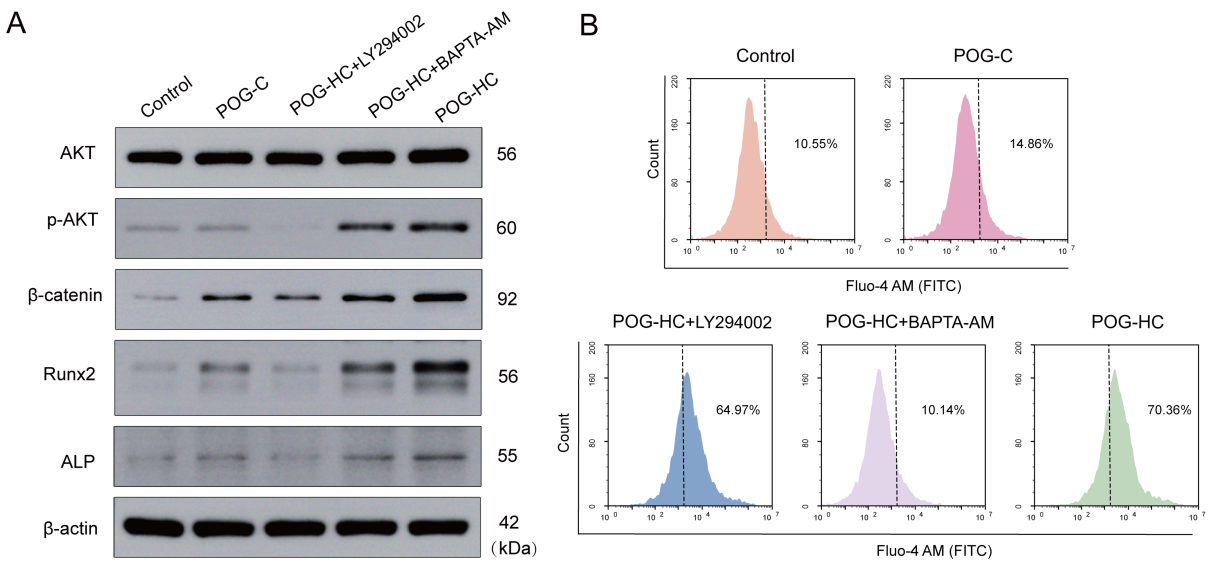


**Figure S16. Changes in osteogenic-related protein expression and intracellular Ca²⁺ levels under perturbation of Ca²⁺ signaling and the PI3K/AKT pathway.** A) Western blot analysis of AKT, p-AKT, β-catenin, and osteogenic markers (Runx2 and ALP) in cells from different treatment groups. B) Intracellular Ca²⁺ levels in each group were measured by flow cytometry after staining with a fluorescent Ca²⁺ probe.

**Materials and methods**

1. **Synthesis and Characterization of ZnO/ZnS heterojunction and POG-HC Hydrogels**
   1. **Synthesis of ZnO**

A total of 1.552 g of zinc acetate dihydrate (Zn(CH₃COO)₂·2H₂O; Sigma-Aldrich, Shanghai, China) was dissolved in 40 mL of deionized water under magnetic stirring. Then, 3.2 g of sodium hydroxide (NaOH; Sinopharm Chemical Reagent Co., Ltd Shanghai, China) was added to the solution and stirred at room temperature for 1 h. The resulting mixture was transferred to a 100 mL autoclave and maintained at 100 °C for 24 h in a convection oven. After the reaction, the white precipitate was collected by centrifugation and washed three times alternately with deionized water and ethanol via redispersion and filtration. The purified product was dried at 80 °C for 10 h and denoted as ZnO.

- 1. **Synthesis of O_V_-rich ZnO/ZnS heterojunction**

In a typical procedure, 0.1 g of the as-prepared ZnO powder was dispersed in 70 mL of deionized water and stirred for 30 min. Subsequently, various amounts of thioacetamide (C₂H₅NS, 0.02–0.1 g; Sigma-Aldrich, Shanghai, China) were added to the solution and stirred for an additional 30 min. The mixture was then transferred to a 100 mL stainless-steel autoclave and heated at 180 °C for 24 h to induce the surface sulfurization reaction. Subsequently, the obtained samples were washed alternately with water and ethanol three times and dried at 80°C for 12 hours to obtain the precursor material. The obtained precursor material was placed in a Chemical Vapor Deposition (CVD) system and calcined at 400°C for 2 h under an Ar atmosphere to yield the ZnO/ZnS heterojunction materials.

Based on the mass of C_2_H_5_NS, the prepared heterojunction materials were named ZnO/ZnS-x (x=0.02, 0.04, 0.06, 0.08 and 0.1) accordingly.

- 1. **Synthesis of OCMC-Na**

2 g of sodium carboxymethyl cellulose (CMC-Na; Leyan.com, Shanghai Haohong Biomedical Technology Co., LTD, Shanghai, China) was gradually added to 40 mL of water and stirred until homogeneous. Meanwhile, 1.1 g of sodium periodate (NaIO_4_; Sinopharm Chemical Reagent Co., Ltd Shanghai, China) was dissolved in 20 mL of water and stirred in the dark to ensure complete mixing. The NaIO_4_ solution was then added to the CMC-Na solution, followed by the addition of 200 μL of hydrochloric acid (HCl; Sinopharm Chemical Reagent Co., Ltd Shanghai, China), and the mixture was stirred in the dark for 5 hours. Subsequently, excess ethanol was added to precipitate the product. The resulting white precipitate was washed with ethanol and centrifuged, with this process repeated three times. The final product was dried overnight in an oven at 35°C to obtain OCMC-Na, which was then stored in a refrigerator for preservation.

- 1. **Synthesis of POG-HC hydrogels**

0.294 g of sodium citrate dihydrate (Na_3_Ct·2H_2_O; Sinopharm Chemical Reagent Co., Ltd Shanghai, China) was dissolved in 3 mL of deionized water, followed by the addition of 0.1 g of gelatin (Gel; Sigma-Aldrich, Shanghai, China). The mixture was stirred at 50°C for 20 minutes to obtain Solution A, which was then set aside for further use.

Next, 0.09 g of magnesium chloride (MgCl_2_; Sigma-Aldrich, Shanghai, China) was dissolved in 6 mL of deionized water, and 0.1 g of OCMC-Na and 0.05 g ZnO/ZnS_-0.04_ was added. The mixture was stirred at 60°C until fully dissolved. Then, 0.9 g of polyvinyl alcohol (PVA, 98.0-99.0 mol% alcoholysis degree, 54.0-66.0 mPa·s viscosity; Sigma-Aldrich, Shanghai, China) was added, and the solution was heated in an oil bath at 90°C for 1 hour. After cooling in water to remove air bubbles, Solution B was obtained.

​Solution A was heated at 90°C for 10 minutes and then added to Solution B. The combined solution was continuously stirred at 90°C for 20 minutes, followed by 15 minutes of sonication to eliminate bubbles, yielding the POG precursor solution. The precursor solution was poured into a Petri dish and frozen at -20 °C for 24 h to form the POG-HC hydrogel.

The synthesis procedures for POG and POG-H followed the same protocol, with the following modifications: POG contained neither ionic salts nor heterojunctions, while POG-H incorporated heterojunctions but excluded ionic salts.

- 1. **Characterization and Electrochemical Measurements**

​X-ray diffraction (XRD) analysis was performed using a Rigaku SmartLab SE diffractometer (Japan) with a scanning range of 10–80° and a step rate of 5°/min. X-ray photoelectron spectroscopy (XPS) measurements were conducted using a Thermo Scientific K-Alpha system (USA). Scanning electron microscopy (SEM) images were acquired using a ZEISS Sigma 300 microscope (Germany), while transmission electron microscopy (TEM) was performed on a JEOL JEM-F200 (Japan). Kelvin probe force microscopy (KPFM) was conducted using an SCM-PIT-V2 probe. Electron paramagnetic resonance (EPR) spectra were recorded using a Bruker EMXplus spectrometer at 77 K. Fourier-transform infrared spectroscopy (FTIR) was performed using a Thermo Fisher Scientific Nicolet iS20 spectrometer (USA). All electrochemical measurements were carried out using a CHI760E electrochemical workstation (CH Instruments, USA). Electrochemical Impedance Spectroscopy (EIS) Testing: The hydrogel sample was sandwiched between two stainless steel electrodes, and electrochemical impedance spectroscopy (EIS) was conducted over a frequency range of 10⁶ to 10⁻¹ Hz with an applied AC voltage of 5 mV (0.005 V). The electrochemical conductivity could be calculated by followed formula:

$$\text{σ}\text{=}\frac{\text{L}}{\text{R}\text{×}\text{A}}$$

Parameters:​σ: Conductivity (S/cm)；L: Distance between electrodes (cm)； R: Bulk resistance obtained from EIS fitting (Ω)； A: Contact area between the electrode and hydrogel (cm²).

1. **Cell Sources and Ethics Statement**

Human periodontal ligament stem cells (hPDLSCs; RRID not available) were purchased from Fuheng Biology (Shanghai, China; Cat. No. FH-H103) and used at passages 3–6. These primary cells were cultured in alpha minimum essential medium (α-MEM; Gibco, USA) supplemented with 10% fetal bovine serum (FBS; Gibco) and 1% penicillin–streptomycin (Beyotime, China) at 37 °C in a humidified atmosphere of 5% CO₂. The murine macrophage cell line **RAW264.7** (RRID:CVCL_0493) was obtained from the **Cell Bank of the Chinese Academy of Sciences** (Cat. No. SCSP-5036, Shanghai, China) and maintained in Dulbecco’s modified Eagle medium (DMEM; Gibco, USA) containing **10% FBS** and **1% penicillin–streptomycin** under the same conditions. All cells were routinely tested and confirmed to be mycoplasma-free prior to use. Cell culture procedures were performed under aseptic conditions, with the culture medium replaced every 2–3 days. All experimental animals were provided by **Shanghai Shengchang Biotechnology Co., Ltd.**; all **animal procedures** were approved by the **Animal Ethics and Use Committee** of Shanghai Shengchang Biotechnology Co., Ltd. (**Ethics No. 2025-01-KOYY-WXL-126**).

1. **Cytotoxicity Evaluation of Hydrogels *In Vitro***
   1. **Live/Dead Cell Viability Staining**

Cytotoxicity of the piezoelectric hydrogels was assessed using a Calcein-AM/PI Live/Dead assay kit (Dojindo, Japan). Hydrogels were pre-sterilized by immersion in 2% (v/v) penicillin–streptomycin for 24 h, UV irradiation for 30 min, and thorough PBS rinsing. An indirect Transwell co-culture (Corning Costar, USA) was used: hPDLSCs (1 × 10⁵ cells/well) were seeded in the lower chamber and hydrogels were placed in the inserts. After 48 h, cells were stained following the manufacturer’s instructions and imaged by inverted fluorescence microscopy (Leica DMi8, Germany). Live (green) and dead (red) cells were quantified using Image J.

- 1. **Hemolysis Assay**

Hemolytic behavior of the hydrogels was evaluated by quantifying free hemoglobin in the supernatant after red blood cell (RBC) exposure. Briefly, whole blood (5 mL) was collected from Sprague–Dawley (SD) rats, and RBCs were isolated by centrifugation (1500 rpm, 10 min). The RBC pellet was washed repeatedly with physiological saline until the supernatant became clear; the cells were then resuspended in saline to prepare a 5% (v/v) RBC suspension. Next, hydrogel samples from each group were extracted in phosphate-buffered saline (PBS) (100 mg/mL; 10% w/v) at 37°C for 24 h. Equal volumes of the hydrogel extract and the RBC suspension (0.6 mL each) were mixed and incubated at 37°C for 2 h. PBS and 1% Triton X-100 served as the negative and positive controls, respectively. After incubation, the mixtures were centrifuged, and the absorbance of the collected supernatant was measured at 540 nm using a microplate reader (BioTek, USA). The hemolysis ratio was calculated using the following formula (the hemolysis ratio < 5% is considered indicative of good hemocompatibility):

$$\text{Hemolysis Rate (\%)=}\frac{\text{(}\text{OD}_{\text{sample}}\text{-}\text{OD}_{\text{negative control}}\text{)}}{\text{(}\text{OD}_{\text{positive control}}\text{-}\text{OD}_{\text{negative control}}\text{)}}\text{×100\%}$$

### ****Cell Adhesion****

Sterilized hydrogels, pre-treated with penicillin–streptomycin solution and UV-irradiated, were placed in 12-well plates for subsequent experiments. hPDLSCs and RAW264.7 cells were seeded onto the hydrogels at a density of 2 × 10⁵ cells/well and co-cultured for 12 h. After incubation, the samples were fixed with 2.5% glutaraldehyde (Servicebio, China), dehydrated through a graded ethanol series, dried, and sputter-coated with gold. Cell adhesion and morphological characteristics on the hydrogel surfaces were observed using scanning electron microscopy (SEM; SEM2000, CIQTEK, China).

### ****Intracellular ROS Detection****

Intracellular ROS levels induced by different hydrogels under ultrasound stimulation were assessed using the DCFH-DA probe (Beyotime, China). Briefly, PDLSCs were seeded in 12-well plates and cultured to an appropriate density, followed by co-culture with the hydrogel samples from each group. Cells were then subjected to ultrasound stimulation (1 MHz, 0.5 W/cm², 15 min). Immediately after stimulation, cells were incubated with DCFH-DA working solution (prepared according to the manufacturer’s instructions) at 37 °C in the dark for 20–30 min. After incubation, cells were washed with PBS and imaged using confocal laser scanning microscopy (green channel) to acquire fluorescence images. For quantitative analysis, cells treated under the same conditions were collected by trypsinization, resuspended in PBS, and analyzed by flow cytometry to determine fluorescence intensity in the FITC channel. To evaluate the reversibility of ROS changes, the POG-HC group was allowed to recover for 60 min after 15 min ultrasound stimulation, and ROS levels were then measured following the same procedure. At least three biological replicates were included for each group.

### ****Transmission Electron Microscopy (TEM) Observation of Mitochondria****

To examine potential ultrastructural changes in mitochondria after ultrasound stimulation, PDLSCs were co-cultured with the hydrogel samples from each group and exposed to ultrasound under the same parameters as above for 15 min, followed by further culture for 24 h. Cells were harvested (trypsinized and pelleted by centrifugation) and fixed with 2.5% glutaraldehyde at 4 °C overnight, then washed with PBS buffer. The samples were subsequently dehydrated through a graded ethanol series, embedded in epoxy resin, and polymerized. Ultrathin sections (~70 nm) were prepared and stained with uranyl acetate and lead citrate. Mitochondrial ultrastructure was observed and imaged using a transmission electron microscope (Hitachi HT-7800, Japan). For each group, multiple fields of view were randomly selected for evaluation, focusing on the integrity of the outer mitochondrial membrane, the clarity of cristae architecture, and typical damage features such as mitochondrial swelling or cristae disruption/loss.

### ****Biosafety Evaluation of Hydrogels *In Vivo*****

In vivo biocompatibility of the hydrogels was assessed using healthy male SD rats (8 weeks old, 240 ± 20 g; n = 10). Animals were anesthetized by intraperitoneal administration of 1% sodium pentobarbital (30 mg/kg). Following dorsal hair removal and antiseptic preparation, sterile POG-HC hydrogel discs (≈10 mm in diameter and ≈2 mm in thickness) were placed into bilaterally symmetric subcutaneous pockets on the back. At 1, 2, 3, and 4 weeks after implantation, the rats were euthanized. The hydrogels and adjacent tissues were harvested, fixed in 4% paraformaldehyde (Servicebio, China), and processed for routine paraffin embedding. Tissue sections were stained with hematoxylin and eosin (H&E) and immunohistochemically stained for IL-1β (1:100, Santa Cruz, Cat No. sc-52012, USA) to assess inflammatory cell infiltration, fibrotic response, and material degradation. In addition, major organs (heart, liver, spleen, lung, and kidney) from rats sacrificed at week 4 were collected, paraffin-embedded, and subjected to H&E staining to evaluate potential histopathological abnormalities and systemic toxicity.

1. **Biofunctional Effects of Piezoelectric Hydrogel on hPDLSCs**

### ****EdU Proliferation Assay****

The effect of the piezoelectric hydrogel on hPDLSC proliferation was evaluated using an EdU Cell Proliferation Kit (Beyotime, China). hPDLSCs were seeded into 12-well plates at a density of 5 × 10⁴ cells/well, and sterilized hydrogel samples were placed in each well. Cells were co-cultured with daily ultrasound (US) stimulation (1 MHz, 0.5 W/cm², 15 min) for 48 h. EdU staining was then performed according to the manufacturer’s instructions. Briefly, cells were incubated with EdU working solution for 2 h, fixed with 4% paraformaldehyde for 15 min, and permeabilized with 0.3% Triton X-100 for 15 min. Afterward, cells were incubated with Click reaction solution in the dark for 30 min, followed by Hoechst 33342 nuclear counterstaining for 10 min. Fluorescence images were acquired using an inverted fluorescence microscope (Leica DMi8, Germany). The proportion of EdU-positive cells was quantified from randomly selected fields using Image J software to evaluate DNA synthesis activity.

### ****Transwell Migration Assay****

A Transwell assay (Corning Costar, USA) was used to evaluate the effect of the piezoelectric hydrogel on the migration of hPDLSCs. Briefly, hPDLSCs were suspended at 1 × 10⁵ cells in 500 μL medium and seeded into the upper chamber of the Transwell insert, while hydrogel samples from different groups were placed in the lower chamber. After applying ultrasound stimulation, cells were incubated for 24 h. Non-migrated cells on the upper side of the membrane were gently removed with a cotton swab. Migrated cells on the lower side were fixed with 4% paraformaldehyde and stained with crystal violet. Images were captured under a microscope from randomly selected fields, and migration rate (%) was quantitatively analyzed using Image J software.

### ****ALP Activity Assay****

To evaluate the osteoinductive potential of the hydrogel, a co-culture system of hPDLSCs and hydrogel was established in 12-well plates. hPDLSCs were seeded at a density of 5 × 10⁴ cells/well and cultured until ~80% confluence. Then, the medium was replaced with osteogenic induction medium consisting of α-MEM supplemented with 10 mM β-glycerophosphate, 50 μg/mL L-ascorbic acid, and 10 nM dexamethasone. The medium was refreshed every 3 days. Ultrasound stimulation was applied daily for 7 consecutive days. On day 7, ALP staining was performed using a BCIP/NBT ALP activity Kit (Beyotime, China). After staining, cells were rinsed with distilled water and observed under a light microscope to evaluate the staining intensity and distribution of ALP-positive cells. In parallel, quantitative analysis of ALP activity was performed using an ALP Assay Kit (Beyotime, China). After the enzymatic reaction, absorbance was measured at 405 nm using a microplate reader (BioTek, USA), and ALP activity was expressed based on the OD₄₀₅ values.

- 1. **Alizarin Red S Staining for Calcium Deposition**

To assess the mineralization capacity of the hydrogels during the late stage of osteogenic differentiation, the same co-culture system and ultrasound stimulation parameters described above were applied. hPDLSCs were cultured in osteogenic induction medium for 21 days, followed by Alizarin Red S (ARS) staining (Beyotime, China). Briefly, the medium was removed, and cells were fixed with fixative solution for 20 min, followed by three washes with PBS. ARS staining solution was added and incubated at room temperature for 30 min. Cells were then thoroughly rinsed with distilled water and observed under a light microscope. For quantitative analysis, 10% cetylpyridinium chloride (CPC) solution was used to extract the bound dye. The plate was shaken at room temperature for 20 min until the color was completely eluted. The supernatant was collected, and the absorbance was measured at 562 nm using a microplate reader (BioTek, USA) to evaluate the degree of mineralization.

### ****Immunofluorescence Staining of Osteogenic-Related Proteins****

To evaluate the effect of hydrogels on the expression of osteogenic-related proteins, immunofluorescence staining of hPDLSCs was performed at different time points. Specifically, Runx2 (Beyotime, Cat No. AF2593, China) was detected on day 3; collagen type I (Col-1, Proteintech, Cat No. 67288-1-Ig, China) and osteopontin (OPN, Proteintech, Cat No. 22952-1-AP, China) on day 7; and osteocalcin (OCN, Proteintech, Cat No. 23418-1-AP, China) on day 14. Cells were fixed with 4% paraformaldehyde for 20 min, permeabilized with 0.2% Triton X-100, and blocked with 5% bovine serum albumin (BSA). Subsequently, cells were incubated overnight at 4 °C with the corresponding primary antibodies (dilution 1:200–1:400). After washing, fluorescent secondary antibodies were added and incubated for 1 h at room temperature in the dark. Cell nuclei were counterstained with DAPI, and cytoskeletons were stained with phalloidin. Images were acquired using a confocal laser scanning microscope (Leica STELLARIS, Germany), and fluorescence intensity was quantified using Image J software.

1. **Immunomodulatory Effects of the Hydrogel on RAW264.7 Macrophages**

### ****Stimulation and Treatment of RAW264.7 Macrophages****

RAW264.7 murine macrophages were seeded in 12-well plates at a density of 2 × 10⁵ cells/well and cultured for 24 h. Lipopolysaccharide (LPS; 100 ng/mL, Beyotime, China) was then added to induce M1 polarization for 12 h. After stimulation, the LPS-containing medium was removed, and cells were gently rinsed twice with PBS. Fresh complete medium was then added along with sterilized hydrogel samples from different groups (POG, POG-H, POG-HC). A control group without hydrogel was also included. US stimulation was applied at 1 MHz, 0.5 W/cm² for 15 min, and the cells were incubated for an additional 12 h. At the end of the treatment, cells and culture supernatants were collected for subsequent **immunofluorescence staining**, **flow cytometry**, and **enzyme-linked immunosorbent assay (ELISA)** to evaluate the effect of the hydrogels on macrophage polarization and cytokine secretion.

### ****ELISA**** Assay ****for Inflammatory Cytokine Secretion****

Culture supernatants were collected and centrifuged to remove debris. The levels of inflammatory cytokines were quantified using enzyme-linked immunosorbent assay (ELISA). Specifically, commercial ELISA kits (Abclonal, China) were used to detect murine **TNF-α** (Cat No. RK00027), **IL-6** (Cat No. RK00008), **IL-10** (Cat No. RK00016), and **TGF-β** (Cat No. RK00057). All assays were performed according to the manufacturers’ protocols, with replicate wells set for each group. Absorbance was measured at 450 nm using a microplate reader (BioTek EPOCH2, USA). Standard curves were generated from serial dilutions of known concentrations, and cytokine concentrations were calculated based on the OD values and standard curves, expressed as pg/mL.

### ****Immunofluorescence Analysis of Macrophage Polarization****

After treatment, RAW264.7 cells were fixed with 4% paraformaldehyde for 15 min and washed with PBS. Cells were permeabilized with 0.2% Triton X-100 for 10 min and then blocked with 5% BSA for 1 h at room temperature. The following primary antibodies were applied: anti-F4/80 (1:200, macrophage marker; Proteintech, Cat No. 29414-1-AP, China), anti-CD86 (1:200, M1 marker; Proteintech, Cat No. 26903-1-AP, China), and anti-CD206 (1:500, M2 marker; Proteintech, Cat No. 83485-1-RR, China). Cells were incubated with primary antibodies overnight at 4 °C. After washing with PBS, appropriate fluorescence-conjugated secondary antibodies were added and incubated for 1 h at room temperature in the dark. Cell nuclei were counterstained with DAPI. Images were acquired using a laser scanning confocal microscope (Leica STELLARIS, Germany). Fluorescence intensity was quantified using Image J software to assess the polarization status of macrophages.

### ****Flow Cytometry Analysis****

After treatment, RAW264.7 cells were gently detached with trypsin, collected, and resuspended in cold PBS. The cells were then adjusted to 2 mL using Flow Cytometry Staining Buffer. For surface marker analysis, Cells were stained separately with PE-CD86 (0.2 μg/10⁶ cells, Proteintech, Cat No. PE-65068, China) or PE-CD206 (0.1 μg/10⁶ cells, Proteintech, Cat No. PE-98031, China). A PE-conjugated IgG isotype control (0.2 μg/10⁶ cells, Proteintech, Cat No. PE-98136, China) was used as a negative control. Staining was performed at room temperature in the dark for 30 min following the manufacturer’s recommended working concentrations. After incubation, cells were centrifuged at 350 ×g for 5 min and resuspended in Flow Cytometry Staining Buffer. Data were collected using a flow cytometer (NovoCyte, Agilent, USA), with at least 10,000 events recorded per sample. The percentage of CD86⁺ (M1) and CD206⁺ (M2) cells was analyzed using NovoExpress® software.

1. **Piezoelectric Hydrogel-mediated Indirect Immunoregulation of hPDLSCs via Conditioned Medium**

#### ****Preparation and Collection of Conditioned Medium****

To investigate the indirect immunomodulatory effects of hydrogels on stem cell inflammation via macrophage regulation, a conditioned medium (CM) model was established. RAW264.7 cells were seeded in 12-well plates at a density of 2 × 10⁵ cells/well and cultured for 24 h under standard conditions. LPS (100 ng/mL; Beyotime, China) was then added to induce M1 polarization over a 12 h incubation. Afterward, cells were gently washed twice with PBS to remove residual LPS, followed by the addition of sterile hydrogel samples (POG, POG-H, and POG-HC) along with fresh complete medium. Control groups without hydrogels were also included. All groups were subjected to US stimulation (1 MHz, 0.5 W/cm², 15 min) and incubated for an additional 12 h. The supernatants were then collected, centrifuged at 1000 g for 10 min, and filtered through a 0.22 μm membrane to obtain CM. Prior to use, CM was mixed with α-MEM complete medium at a 1:1 volume ratio and applied to hPDLSCs for 24 h of stimulation.

#### ****Immunofluorescence Analysis of Inflammatory Cytokine Expression****

hPDLSCs were seeded in 24-well plates at 2 × 10⁴ cells/well and allowed to adhere. The medium was then replaced with CM from different treatment groups and incubated for 24 h. Cells were fixed with 4% paraformaldehyde for 15 min, permeabilized with 0.2% Triton X-100 for 10 min, and blocked with 5% BSA for 1 h. Subsequently, cells were incubated overnight at 4 °C with primary antibodies against TNF-α (1:200, Proteintech, Cat No. 17590-1-AP, China) and IL-6 (1:200, Affinity, Cat No. DF6087, USA). The following day, cells were washed and incubated with fluorescently labeled secondary antibodies for 1 h at room temperature in the dark. Nuclei were counterstained with DAPI. Fluorescence images were captured using a confocal laser scanning microscope (Leica STELLARIS, Germany), and cytokine expression levels were quantified using Image J software based on fluorescence intensity.

### ****Western Blot Analysis****

Following CM stimulation, hPDLSCs were lysed on ice for 30 min using RIPA lysis buffer containing protease and phosphatase inhibitors (Beyotime, China). The lysates were centrifuged at 12,000 rpm for 10 min at 4 °C, and the supernatants were collected. Total protein concentrations were determined using a BCA Protein Assay Kit (Beyotime, China). Protein samples were mixed with 5× SDS loading buffer and denatured at 100 °C for 5 min. Equal amounts of protein were separated by sodium dodecyl sulfate-polyacrylamide gel electrophoresis (SDS-PAGE) and transferred onto polyvinylidene difluoride (PVDF) membranes (Millipore, Sigma, USA). After blocking, the membranes were incubated overnight at 4 °C with the following primary antibodies: NF-κB (1:1000, Proteintech, Cat No. 10745-1-AP, China), p-NF-κB (1:5000, HUABIO, Cat No. HA723223, China), TNF-α (1:1000, Proteintech, Cat No. 17590-1-AP, China), and β-Actin (1:10000, Proteintech, Cat No. 81115-1-RR, China). After washing, membranes were incubated with HRP-conjugated secondary antibodies for 1 h at room temperature. Protein bands were visualized using an enhanced chemiluminescence (ECL) detection system (Tanon 4600, China), and the relative expression levels were quantified by densitometry using Alpha software to assess activation of inflammation-related signaling pathways.

### ****Evaluation of Piezoelectric Hydrogel Therapy for Periodontitis *In Vivo*****

#### ****Construction of the Periodontitis Animal Model****

Twenty male SD rats (8 weeks old, 240 ± 20 g) were acclimatized for one week before the experiment. Anesthesia was induced by intraperitoneal injection of 1% sodium pentobarbital (30 mg/kg). Chronic periodontitis was established by ligating the maxillary second molars with 0.2 mm orthodontic stainless-steel wire and injecting 10 μL of LPS solution (100 ng/mL) into the gingival sulcus before ligation. The ligatures were maintained for 4 weeks to develop stable chronic periodontal lesions. After successful model establishment, the animals were randomly assigned to five groups (n = 4): Healthy group: no ligation or LPS injection, serving as a healthy control; Control group: periodontitis induced by ligation, followed by periodontal pocket injection of PBS; POG group: periodontal pocket injection of POG hydrogel; POG-H group: periodontal pocket injection of POG-H hydrogel; POG-HC group: periodontal pocket injection of POG-HC hydrogel. For all treatment groups, the hydrogels were injected into the periodontal pockets once weekly after ligature removal. US stimulation (1 MHz, 0.5 W/cm², 15 min) was applied three times per week. After 4 weeks of treatment, the animals were sacrificed, and maxillary bone specimens were collected for further analysis.

#### ****Micro-CT Imaging and Bone Parameter Analysis****

High-resolution micro-CT (SkyScan 1276, Bruker, USA) was performed to obtain three-dimensional images of the maxillary molar region (scan settings: 9 μm voxel resolution, 50 kV voltage, 200 μA current). After image reconstruction, the mesial root region of the maxillary second molar was selected as the region of interest (ROI) for quantitative bone analysis. The following bone parameters were measured: CEJ–ABC distance, BV/TV, Tb.N, Tb.Sp. Data processing was performed using CTvox and CTAn software (Bruker, USA). The mean values of each parameter per group were calculated for statistical analysis.

### ****Histological and Immunofluorescence Analysis****

Maxillary specimens were fixed in 4% paraformaldehyde for 48 h, decalcified in 10% EDTA, dehydrated through a graded ethanol series, embedded in paraffin, and sectioned at a thickness of 3.5 μm. Hematoxylin and eosin (H&E) staining, Masson’s trichrome staining, and tartrate-resistant acid phosphatase (TRAP) staining (Solarbio, China) were performed to evaluate bone regeneration and osteoclastic activity. Immunofluorescence staining was conducted to assess the reconstruction of the immune microenvironment in the alveolar bone region. After deparaffinization and antigen retrieval, the sections were washed with PBS and blocked with 5% BSA for 1 h. The primary antibodies against M1 macrophage marker iNOS (1:200, Proteintech, Cat No. 18704-1-AP, China) and M2 marker CD206 (1:200, Proteintech, Cat No. 18985-1-AP, China) were incubated overnight at 4 °C. On the following day, fluorescence-conjugated secondary antibodies were added and incubated for 1 h at room temperature in the dark, followed by nuclear counterstaining with DAPI. Images were acquired using a confocal laser scanning microscope and a digital slide scanner. Quantitative analysis of histological features and fluorescence intensity was performed using Image J software to evaluate tissue repair and macrophage polarization in response to different hydrogel treatments.

### ****Mechanistic Investigation of Piezoelectric-Induced Osteogenesis****

### ****RNA Sequencing and Bioinformatic Analysis****

To elucidate the molecular mechanisms by which the hydrogel induces osteogenic differentiation in hPDLSCs, whole-transcriptome RNA sequencing was performed on Control and POG-HC groups (n = 3 biological replicates per group). Total RNA was extracted using TRIzol reagent, and RNA concentration and purity were assessed before library construction. Sequencing was conducted by Shanghai Majorbio Bio-pharm Biotechnology Co., Ltd. (Shanghai, China) on the Illumina NovaSeq X Plus platform. After quality control using fastp, clean reads were aligned to the human reference genome using HISAT2, and differentially expressed genes (DEGs) were identified using the DESeq2 package. The threshold for DEGs was set at |log₂ fold change| ≥ 1 and a false discovery rate (FDR) < 0.05. Principal component analysis (PCA), volcano plots, and heatmaps were used to visualize transcriptional differences. Gene Ontology (GO) and Kyoto Encyclopedia of Genes and Genomes (KEGG) enrichment analyses were performed to identify DEGs associated with osteogenesis, calcium signaling, PI3K-AKT, Wnt/β-catenin, and NF-κB pathways.

### ****Intracellular Ca²⁺ Fluorescence Imaging and Flow Cytometric Analysis****

To assess whether hydrogel mediated stimulation triggers Ca²⁺ influx in hPDLSCs, cells were loaded with the calcium sensitive fluorescent probe Fluo-4 AM (Beyotime, China). After ultrasound stimulation (1 MHz, 0.5 W/cm², 15 min), cells were incubated with the probe at 37 °C for 30 min, followed by two PBS washes to remove excess dye. Fluorescence signals were captured using an inverted fluorescence microscope (Leica DMi8, Germany) with an excitation wavelength of 488 nm and an emission wavelength of 516 nm. Fluorescence intensity was quantified using Image J software to estimate changes in intracellular free Ca²⁺ levels. In addition to fluorescence imaging, intracellular Ca²⁺ levels were quantitatively analyzed by flow cytometry. Briefly, Fluo-4 AM–loaded cells treated under the same conditions were harvested by trypsinization, resuspended in PBS, and analyzed in the FITC channel to determine mean fluorescence intensity. To further confirm the specificity of Ca²⁺ signaling, an intracellular Ca²⁺ chelator, BAPTA-AM (MedChemExpress, HY-100545), was included as an inhibitor control group to buffer cytosolic Ca²⁺ and assess its contribution to the observed responses.

### ****Western Blot Analysis of Osteogenesis-Related Pathways****

Western blotting was conducted to validate protein-level changes in osteogenic and signaling markers. Total proteins were extracted using RIPA lysis buffer supplemented with protease and phosphatase inhibitors. After quantification and denaturation, samples were separated via SDS-PAGE and transferred to PVDF membranes. The membranes were blocked and probed overnight at 4 °C with the following primary antibodies: AKT (1:2000, Abcam, ab185633), p-AKT (1:2000, CST, 4060S), Runx2 (1:1000, Beyotime, AF2593), ALP (1:300, Santa Cruz, sc-365765), β-catenin (1:1000, Abcam, ab32572), and β-Actin (1:10000, Proteintech, 81115-1-RR) as the internal control. After incubation with HRP-conjugated secondary antibodies for 1 h at room temperature, protein bands were visualized using an ECL detection system (Tanon 4600, China). Band intensity was quantified using Alpha software to determine the relative expression levels and assess pathway activation and osteogenic potential. To further dissect the underlying signaling mechanisms, pathway-perturbation controls were introduced in parallel. Specifically, hPDLSCs treated with POG-HC were co-incubated with either the PI3K inhibitor LY294002 (MedChemExpress, HY-10108) or the intracellular Ca²⁺ chelator BAPTA-AM (MedChemExpress, HY-100545), denoted as the POG-HC+LY294002 and POG-HC+BAPTA-AM groups, respectively. These inhibitor-intervention groups were used to evaluate the functional contribution of PI3K/AKT signaling and cytosolic Ca²⁺ dynamics to osteogenic pathway activation, as reflected by changes in p-AKT, β-catenin, Runx2, and ALP expression.
